# Addition of two new genera—*Marcstadlera* gen. nov. and *Neoclypeosphaerella* gen. nov. (*Mycosphaerellaceae*)—based on polyphasic evidence

**DOI:** 10.3389/fcimb.2025.1668928

**Published:** 2025-10-23

**Authors:** Gargee Singh, Soumyadeep Rajwar, Sahana Khatoon, Sanjay Yadav, Pooja Kumari, Raghvendra Singh, Kamalesh Kumar, Smriti Mall, Paras N. Singh, Shambhu Kumar, Uwe Braun

**Affiliations:** ^1^ Department of Botany, DDU Gorakhpur University, Gorakhpur, Uttar Pradesh, India; ^2^ Centre of Advanced Study in Botany, Institute of Science, Banaras Hindu University, Varanasi, Uttar Pradesh, India; ^3^ School of Life Sciences, Jawaharlal Nehru University, New Delhi, India; ^4^ Department of Chemistry, Institute of Science, Banaras Hindu University, Varanasi, Uttar Pradesh, India; ^5^ National Fungal Culture Collection of India (NFCCI), Biodiversity and Palaeobiology Group, MACS Agharkar Research Institute, Pune, Maharashtra, India; ^6^ Forest Pathology Department, KSCSTE-Kerala Forest Research Institute, Thrissur, Kerala, India; ^7^ Martin-Luther-Universität, Institut für Biologie, Bereich Geobotanik and Botanischer Garten, Herbarium, Halle (Saale), Germany

**Keywords:** anamorph, *Dothideomycetes*, multigene-phylogeny, *Mycosphaerellales*, new taxa, nomenclature

## Abstract

During a survey of foliicolous fungi in India, two interesting anamorphic hyphomycetous fungal specimens were collected from infected leaves of *Calotropis* spp. and *Mallotus philippensis*. *Calotropis* spp. produce fascicles of conidiophores from stromata, accompanied by secondary superficial hyphae bearing solitary conidiophores. The specimen on *Mallotus philippensis* resembled *Mycovellosiella*, characterized by secondary superficial hyphae bearing micronematous to semi-macronematous, mononematous, unbranched, and aseptate conidiophores. A polyphasic approach—including morphological, cultural, and multilocus phylogenetic analyses (LSU-*Rpb2*-ITS), coupled with genealogical concordance phylogenetic species recognition—identified its relationship with cercosporoid fungi within the family *Mycosphaerellaceae*. The analysis confirmed that these fungal specimens represent distinct lineages without known morphological or DNA sequence counterparts. Consequently, two new genera are proposed: *Marcstadlera* and *Neoclypeosphaerella*, with *M. malloti* comb. nov. and *N. calotropidis* comb. nov. as their respective type species. Additionally, *Clypeosphaerella calotropidis*, *Clypeosphaerella quasiparkii*, and *Pseudocercospora malloti* are recognized as new synonyms. Several genera in the *Mycosphaerellaceae*, including *Marcstadlera* and *Neoclypeosphaerella*, are monophyletic. The ultrastructure of the conidiogenous loci and hila differs between these two genera. In *Marcstadlera*, the loci are cylindrical or peg-like, truncate at the apex, while the conidial base is narrowly obconically truncate. In *Neoclypeosphaerella*, the loci are slightly protuberant and surrounded by a circular rim-like structure, forming a truncated apex with a centrally positioned small apical depression. The conidial base is obconically truncated and also surrounded by a circular rim-like structure.

## Introduction

*Mycosphaerellaceae* Lindau is a diverse family of fungi in the order *Mycosphaerellales* (*Ascomycota*), comprising over 3,000 species (Crous and Braun, 2003; Braun et al., 2013, Braun et al., 2015, Braun et al., 2016). They thrive in diverse habitats and exhibit diverse life modes, including pathogenic, endophytic, saprophytic, and epiphytic modes of existence in various hosts worldwide (Videira et al., 2017). They have garnered significant research attention due to their association with a wide range of economically and ornamentally important host plants (Videira et al., 2017; Abdollahzadeh et al., 2020; Bakhshi et al., 2021; Bakhshi and Braun, 2022).

Members of *Mycosphaerellaceae* exhibit both sexual (teleomorphic) and asexual (anamorphic) stages (Crous et al., 2009). However, many, or perhaps most, species in this complex are asexual holomorphs—that is, they have lost the ability to produce sexual morphs. Within this complex, the sexual morphs of *Mycosphaerella* are morphologically quite uniform and offer few distinguishing features to justify further division into smaller genera. In contrast, the asexual morphs exhibit significant morphological diversity, which has led to the establishment of numerous asexual genera based on specific morphological traits (Videira et al., 2017).

Currently, more than 120 genera have been accepted in *Mycosphaerellaceae* (Wijayawardene et al., 2014; Videira et al., 2017; Crous et al., 2020b; Bakhshi et al., 2020, Bakhshi and Braun, 2022; Yadav et al., 2022, Yadav et al., 2023; Bakhshi and Crous, 2024; Melo et al., 2025). Advances in molecular phylogenetics have significantly reshaped the taxonomy of this family, uncovering cryptic species and refining classification (Crous et al., 2007, Crous et al., 2013a, Crous et al., 2013b; Verkley et al., 2013; Quaedvlieg et al., 2014; Videira et al., 2017; Bakhshi et al., 2021; Bakhshi and Braun, 2022). Beyond their pathogenic roles, *Mycosphaerellaceae* species play a crucial part in ecological dynamics, influencing plant health and ecosystem stability.

*Clypeosphaerella* Guatim. et al. and *Mycovellosiella* Rangel are the two notable genera within the *Mycosphaerellaceae*, and the members of these genera typically cause leaf spot diseases. The genus *Clypeosphaerella* exhibits both sexual and asexual morphs. The sexual morph is distinguished by its thicker upper ascomatal wall, resembling a pseudoclypeus. In contrast, the asexual morph develops fasciculate conidiophores from stromata, as well as solitary conidiophores arising from secondary superficial hyphae, with conidia forming singly or in chains (Chupp, 1954; Kamal et al., 1990; Braun, 2000a; Wilkinson et al., 2005; Haldar and Ray, 2001; Guatimosim et al., 2016; Videira et al., 2017). Similarly, the genus *Mycovellosiella* is characterized by the absence or poor development of stromata. It produces secondary superficial hyphae that give rise to solitary or fasciculate conidiophores as lateral branches, with conidia forming either singly or in chains (Videira et al., 2017).

During a 2023–2024 survey of foliicolous fungi in Uttar Pradesh, India, two anamorphic hyphomycetous fungal specimens were collected from diseased leaves. The first specimen was found on *Calotropis* spp., where it developed fascicles of conidiophores from stromata, accompanied by secondary superficial hyphae bearing solitary conidiophores. Molecular phylogenetic analyses revealed that the isolate forms an independent lineage within *Mycosphaerellaceae*, clustering with *Clypeosphaerella calotropidis* but remaining distinct from *C. sticheri*, the type species of *Clypeosphaerella*. To accommodate this unique lineage, the novel genus *Neoclypeosphaerella* is proposed, emphasizing its uniqueness within *Mycosphaerellaceae*.

Similarly, another specimen, *Mycovellosiella malloti*—the basionym of *Pseudocercospora malloti*—was isolated from *Mallotus philippensis*, where it developed secondary superficial hyphae with micronematous to semi-macronematous solitary conidiophores. Phylogenetic analyses revealed that this isolate segregates from *Mycovellosiella*, forming an independent lineage within *Mycosphaerellaceae*. As a result, the novel genus *Marcstadlera* is proposed to accommodate this fungus, underscoring its distinct evolutionary trajectory within the family. *Mycovellosiella* was previously distinguished from closely related genera, *Passalora* Fr. and *Phaeoramularia* Munt.-Cvetk. based on the formation of superficial mycelium with solitary conidiophores formed *in vivo*. However, these traits are phylogenetically and taxonomically insignificant and appear unreliable (Videira et al., 2017). Therefore, species exhibiting mycovellosiella-like morphology should be tentatively maintained in or assigned to *Passalora s. lat.*, unless their phylogenetic affinity is thoroughly investigated (Videira et al., 2017).

These taxonomic revisions, driven by molecular phylogenetics and morphological analyses, refine the classification and relationships of these taxa and are discussed in detail in this manuscript.

## Materials and methods

### Sample collection and fungal isolation

During a field survey of phytopathogenic fungi conducted between 2023 and 2024, infected leaves of *Calotropis* spp. were collected from Kushmi Forest, while infected leaves of *Mallotus philippensis* were collected from Varanasi and Mirzapur, Uttar Pradesh, India. These locations are part of tropical dry deciduous forest ecosystems, primarily consisting of dense forests dominated by Sal and Teak trees. This region is characterized by hot, dry summers, mild winters, undulating hilly terrain, and rain-fed streams. Herbs and shrubs are commonly found during the rainy season. The collected infected leaves were placed in sterilized polybags along with relevant collection details and transported to the laboratory for further processing. Standard techniques as described by Hawksworth (1974); Savile (1962), and Verma et al. (2021c) were followed. In the lab, the samples were dried for approximately 1 week between fresh sheets of blotting paper. The dried and pressed leaf specimens were then sealed in airtight polyethylene bags and stored in paper envelopes, accompanied by the corresponding collection information.

Slides were mounted in a 1:1 mixture of glycerine and lactophenol cotton-blue from the infected part of the leaves. Observations were made with a stereo zoom microscope (Magnus: MSZ-TR) with an attached camera (CatCam300EF) and an Olympus compound microscope (BX53) equipped with differential interference contrast (DIC) illumination, and images were captured using an Olympus DP28 camera with associated software. Scanning electron microscopy (SEM) was conducted using a field emission scanning electron microscope (FEI Nova Nano SEM-450). For SEM micrographs, specimens were coated with gold-palladium using a POLARON Sputter coater and examined with a LEO-430 scanning electron microscope. Detailed observations of morphological characters were carried out at different magnifications through light microscopy (×450 and ×1,000) and scanning electron microscopy (up to ~×55K). Size ranges of morphological features were determined from at least 25 measurements, and 95% confidence intervals were calculated for the measurements, with the extreme values given in parentheses. The examined reference specimens were deposited in the fungarium of Ajrekar Mycological Herbarium (AMH), MACS, Agharkar Research Institute (ARI), Pune, India, and duplicates were retained in the Mycological Herbarium of the Department of Botany of Banaras Hindu University, Varanasi, U.P., India (MH-BHU). For *in vitro* isolation, conidia were transferred to Petri dishes containing malt extract agar (MEA), potato dextrose agar (PDA), and agar media supplemented with undefined vegetable peelings. The petri dishes were incubated at 25°C ± 5°C and diffused daylight. The ex-type living cultures were deposited at the National Fungal Culture Collection of India (NFCCI), MACS, Agharkar Research Institute, Pune, India.

### DNA extraction, PCR, and sequencing

The genomic DNA was extracted from mycelia and conidia freshly scraped from PDA plates using a sterile scalpel blade. Approximately 200 mg of wet weight was transferred to 2-mL microcentrifuge tubes kept in liquid nitrogen for 2 min and then grinded to a fine powder using a pestle and mortar. DNA was extracted using a modified CTAB method using the protocol of Van Burik et al. (1998). The internal transcribed spacer (ITS) region was amplified using ITS1/ITS4 (White et al., 1990), large subunit nuclear ribosomal DNA (LSU) gene with LROR/LR7 (Vilgalys and Hester, 1990; Rehner and Samuels, 1994), and partial DNA-directed RNA polymerase II subunit (*Rpb2*) with RPB2-5F2/RPB2-7cR (Liu et al., 1999; Sung et al., 2007) primer pairs. Amplification reaction mixtures and conditions described by Yadav et al. (2022, 2023) were followed for standard amplification and subsequent sequencing of the ITS, LSU, and *Rpb2* by Eurofins Genomics (Bengaluru, India).

### Sequence alignment and phylogenetic analysis

The obtained ITS, LSU, and *RBP2* sequences from the isolates NFCCI 5818, NFCCI 5819, NFCCI 5983, and NFCCI 5984 were assembled and edited using Chromas v.2.6.6. The manually edited sequences were submitted to NCBI GenBank (**Table 1**) and were subjected to a megablast search of the NCBI GenBank nucleotide database to retrieve the most closely matched sequences of related strains. Reference sequences were also selected from relevant published literature (**Table 1**). Sequence alignments were generated using MAFFT v.7 (Katoh et al., 2019). The alignments of individual loci were concatenated using Mesquite v. 3.61 (Maddison and Maddison, 2018) and deposited as electronic supplementary materials in TreeBASE (http://www.treebase.org/), under the accession number 32049 and URL http://purl.org/phylo/treebase/phylows/study/TB2:S32049?x-access-code=b06647353af0bee2d49542a8bb895832&format=html.

**Table 1:** Taxa included in the molecular phylogenetic analyses and their GenBank accession numbers.

Taxa	Isolates/ voucher ID	Genbank accession numbers			Host	Country	References
		ITS	LSU	*Rpb2*			
*Acervuloseptoria* *ziziphicola*	CBS 138009/ CPC 23707	KJ869164	KJ869221	MF951425	*Ziziphus mucronata*	South Africa	Crous et al. (2014); Videira et al. (2017)
*Apseudocercosporella trigonotidis*	CPC 10865	KX287276	KX286964	KX288413	*Trigonotis peduncularis*	South Korea	Videira et al. (2016)
*Cercospora apii*	CBS 116455/ CPC 11556	AY840519	MF951133	–	*Apium graveolens*	Germany	Videira et al. (2017)
*Cercospora fagopyri*	CBS 132623/ CPC 14541	JX143594	MF951143	MF951463	*Fagopyrum esculentum*	Republic of Korea	Videira et al. (2017)
*Cercospora sojina*	CBS 132615/ CPC 11353	JX143659	KX286969	KX288419	*Glycine soja*	Republic of Korea	Videira et al. (2017)
*Cercosporella pfaffiae*	Vic31849	JQ990331	–	–	*Pfaffia glomerata*	Brazil	MaChado et al. (2012)
*Cercosporella virgaureae*	CPC 19492	KX287288	KX286981	KX288431	*Conyza canadensis*	Brazil	Videira et al. (2016)
*Cercosporidium chaetomium*	CBS 142177/ CPC 18624	MF951306	MF951151	MF951474	*Euphorbia* sp.	Canada	Videira et al. (2017)
*Cercosporidium miurae*	CPC 14643	KJ633264	KJ633268	MF951473	*Metaplexis japonica*	Republic of Korea	Videira et al. (2017)
*Cercosporidium miurae*	CBS 142235	MF951305	MF951150	MF951472	*Metaplexis japonica*	Republic of Korea	Videira et al. (2017)
*Clypeosphaerella calotropidis*	BRIP 39358	AY303969	–	–	*Calotropis procera*	Australia	Wilkinson et al. (2005)
*Clypeosphaerella calotropidis*	CBS 129.30	MF951308	MF951153	MF951477	*Calotropis procera*	Egypt	Videira et al. (2017)
*Clypeosphaerella quasiparkii*	CBS 123243/ CPC 15409	KF901771	KF902128	MF951478	*Eucalyptus* sp.	Thailand	Videira et al. (2017)
*Clypeosphaerella sticheri*	CPC 24705	KT037546	KT037588	–	*Sticherus bifidus*	Brazil	Guatimosim et al. (2016)
*Clypeosphaerella sticheri*	CPC 24733	KT037536	KT037577	–	*Sticherus bifidus*	Brazil	Guatimosim et al. (2016)
*Coremiopassalora eucalypti*	CBS 111318/ CPC 1457	GU269845	GU253860	MF951482	*Eucalyptus saligna*	Brazil	Videira et al. (2017)
*Coremiopassalora leptophlebae*	CBS 129524/ CPC 18480	MF951310	KF901939	MF951483	*Eucalyptus leptophleba*	Brazil	Videira et al. (2017)
*Distocercospora pachyderma*	CBS 138247/ CPC 24144	MF951311	MF951156	MF951486	*Dioscorea* sp.	Japan	Videira et al. (2017)
*Filiella pastinacae*	CBS 114116/ UPSC 2633	KF251328	KF251832	KX348056	*Laserpitium latifolium*	Sweden	Videira et al. (2017)
*Fusoidiella anethi*	CBS 296.32	MF951318	MF951164	MF951499	–	Italy	Videira et al. (2017)
*Fusoidiella anethi*	CBS 117584	MF951319	MF951165	MF951500	*Foeniculum vulgare*	New Zealand	Videira et al. (2017)
*Fusoidiella depressa*	CBS 141335/ CPC 14915	KF251309	KF251813	KX348055	*Angelica gigas*	Republic of Korea	Videira et al. (2017)
*Graminopassalora geissorhizae*	CBS 146788/ CPC 38623	MW175336	MW175376	MW173111	*Geissorhiza* sp*lendidissima*	South Africa	Crous et al. (2020a)
*Graminopassalora graminis*	CBS 113303	GU214666	GU214666	MF951502	*Alopecurus aequalis* var. *amurensis*	Republic of Korea	Videira et al. (2017)
***Marcstadlera malloti***	**NFCCI 5818**	**PQ012587**	**PQ012588**	**PQ034553**	***Mallotus philippinensis***	**India**	**This study**
***Marcstadlera malloti***	**NFCCI 5819**	**PQ013688**	**PQ013689**	**PQ034554**	***Mallotus philippinensis***	**India**	**This study**
*Mycovellosiella cajani*	CBS 113998/ CPC 5335	KF251315	KF251819	MF951527	*Cajanus cajan*	South Africa	Videira et al. (2017)
*Mycovellosiella cajani*	CBS 113999/ CPC 5339	KF251316	KF251820	MF951528	*Cajanus cajan*	South Africa	Videira et al. (2017)
*Mycovellosiella cajani*	CBS 114275/ CPC 5334	KF251317	KF251821	MF951529	*Cajanus cajan*	South Africa	Videira et al. (2017)
*Neoacervuloseptoria fraxini*	CPC 36558/ CBS 145992	MT223773	MT223870	MT223673	*Fraxinus* sp.	Russia	Crous et al. (2020b)
*Neocercospora ammicola*	CBS 136450/ CCTU 1186	KR232407	KR232405	KX288446	*Ammi majus*	Iran	Videira et al. (2017)
*Neocercosporella peristrophes*	AMH 9671	MZ311866	MZ311874	OL773683	*Peristrophe bicalyculata*	India	Yadav et al. (2023)
*Neocercosporella peristrophes*	AMH 10363	ON310831	ON310846	ON376994	*Peristrophe bicalyculata*	India	Yadav et al. (2023)
***Neoclypeosphaerella calotropidis***	**NFCCI 5983**	**PV112567**	**PQ816342**	**PV125517**	***Calotropis gigantea***	**India**	**This study**
***Neoclypeosphaerella calotropidis***	**NFCCI 5984**	**PV112568**	**PQ816341**	**PV125518**	***Calotropis procera***	**India**	**This study**
*Neopseudocercospora* *terminaliae*	CBS 136423/ CPC 22686	KF777175	KF777228	MF951630	*Terminalia* sp.	Zambia	Videira et al. (2017)
*Neopseudocercosporella* *brassicicola*	CBS 163.26	MF951337	MF951192	MF951548	–	–	Videira et al. (2017)
*Neopseudocercosporella* *brassicicola*	CBS 228.32	KF251304	KF251808	KX348058	*Brassica oleracea*	Denmark	Videira et al. (2017)
*Neopseudocercosporella* *capsellae*	CBS 112032/ HJS 601	KF251320	KF251824	KX348060	*Brassica* sp.	–	Videira et al. (2017)
*Neopseudocercosporella* *capsellae*	CBS 112033/ HJS 600	KF251306	KF251810	KX348061	*Brassica* sp.	–	Videira et al. (2017)
*Neoramulariopsis catenulata*	CBS 355.73	KX287281	KX286973	KX288424	*Phaseolus vulgaris*	Rwanda	Videira et al. (2016)
*Neoramulariopsis unguis-cati*	CBS 138101/ CPC 22948	KJ869140	KJ869197	KX288423	*Dolichandra unguis-cati*	South Africa	Crous et al. (2014); Videira et al. (2016)
*Pedrocrousiella pongamiae*	NFCCI 4881	MW327548	MW327593	MW363496	*Pongamia pinnata*	India	Rajeshkumar et al. (2021)
*Pseudocercospora abacopteridicola*	CPC 24709	KT037518	KT037559	–	*Adiantum* sp.	Brazil	Guatimosim et al. (2016)
*Pseudocercospora abeliae*	MUCC1674	LC599330	–	LC599587	*Abelia chinensis*	Japan	Chen et al. (2022)
*Pseudocercospora airliensis*	BRIP 58550	KM055429	KM055433	–	*Polyalthia nitidissima*	Australia	Shivas et al. (2015)
*Pseudocercospora aleuritis*	MAFF 237174/MUCC 1230	LC599331	–	LC599588	*Aleuritis montana*	Japan	Chen et al. (2022)
*Pseudocercospora convoluta*	CBS 113377	DQ676519	MF951226	MF951617	*Chromolaena odorata*	Costa Rica	Videira et al. (2017)
*Pseudocercospora eucalyptorum*	CBS 114866	KF901720	JQ739817	MF951618	*Eucalyptus nitens*	South Africa	Videira et al. (2017)
*Pseudocercospora vitis*	CPC 11595	GU269829	GU214483	KX348076	*Vitis vinifera*	South Korea	Videira et al. (2017)
*Pteridopassalora lygodii*	BCRC FU20503	KR527201	–	–	*Lygodium japonicum*	Taiwan	Kirschner and Wang (2015); Chen et al. (2022)
*Pteridopassalora nephrolepidicola*	CBS 128211/ CPC 17049	HQ599590	HQ599591	KX462646	*Nephrolepis falcata*	Australia	Crous et al. (2010); Nakashima et al. (2016); Chen et al. (2022)
*Ramichloridium apiculatum*	CBS 156.59/ ATCC 13211/ IMI 100716/ JCM 6972/ MUCL 15753/MUCL 7991/ QM 7716	EU041791	EU041848	MF951416	Forest soil	USA	Videira et al. (2017)
*Ramulariopsis cnidoscoli*	CPC 18242	KX287543	KX287246	KX288705	*Gossypium barbadense*	Brazil	Videira et al. (2016)
*Ramulariopsis gossypii*	CBS 141099/ CPC 25909	KX287540	KX287243	KX288702	*Gossypium* sp.	Brazil	Videira et al. (2016)
*Ramulariopsis gossypii*	RA17.5	KR265337	–	–	Cotton	Brazil	Mehta et al. (2016)
*Rosenscheldiella brachyglottidis*	PDD 94939	GQ355335	GQ355334	–	*Brachyglottis repanda*	New Zealand	Sultan et al. (2011)
*Septoria dysentericae*	CBS 131892/ CPC 12328	GU269854	GU253866	KX348088	*Inula britannica*	South Korea	Crous et al. (2013a); Videira et al. (2016)
*Septoria urticae*	CBS 102375	KF251583	JN940675	MF951668	*Urtica dioica*	Netherlands	Videira et al. (2017)
*Sonderhenia eucalypticola*	CMW 20333	DQ267593	DQ267574	–	*Eucalyptus globulus*	Chile	Hunter et al. (2006)
*Sonderhenia eucalypticola*	CMW 20334	DQ267594	DQ267575	–	*Eucalyptus globulus*	Chile	Hunter et al. (2006)
*Sonderhenia eucalyptorum*	CBS 120220	DQ923536	DQ923536	MF951673	*Eucalyptus coccifera*	Australia	Summerbell et al. (2006); Videira et al. (2017)
*Sonderhenia eucalyptorum*	CPC 17677	MN162019	MN162214	–	*Eucalyptus* sp.	Australia	Crous et al. (2019)
*Sonderhenia* sp.	CPC 17710	MN162025	MN162215	–	*Eucalyptus regans*	Australia	Crous et al. (2019)
*Sphaerulina azaleae*	CBS 128605	MH865035	KF252104	–	*Rhododendron* sp.	South Korea	Vu et al. (2019); Verkley et al. (2013)
*Sphaerulina rhododendricola*	CBS 136435/ CPC 21813	KF777187	KF779493	–	*Rhododendron* sp.	Thailand	Crous et al. (2013b)
*Uwebraunia australiensis*	CBS 120729/ CPC 13282	KF442513	KF442553	KX348105	*Eucalyptus platyphylla*	Australia	Videira et al. (2017)
*Uwemyces elaeidis*	CPUwZC-01	KX228299	KX228356	KX228371	*Elaeis oleifera*	Colombia	Videira et al. (2017)
*Zasmidium cellare*	CBS 146.36	EU041821	EU041878	MF951693	Wall in wine cellar	–	Videira et al. (2017)
*Zasmidium citrigriseum*	CBS 122455	KF901792	KF902151	MF951695	*Citrus* sp.	USA	Videira et al. (2017)
*Zasmidium citrigriseum*	GUCC 1507.3	MT683372	MT712179	MT700485	–	–	An et al. (2021)
*Zasmidium elaeocarpi*	CBS 142187	MF951398	MF951263	MF951699	*Elaeocarpus kirtonii*	Australia	Videira et al. (2017)
*Zasmidium elaeocarpi*	CPC 16640	MF951399	MF951264	MF951700	*Elaeocarpus kirtonii*	Australia	Videira et al. (2017)
*Zasmidium iteae*	CBS 113094	MF951405	MF951271	MF951711	*Itea parviflora*	Taiwan	Videira et al. (2017)

The sequences in bold were generated in this study. “–” indicates missing or unavailable sequences.

Phylogenetic trees were constructed using Bayesian inference (BI) performed with MrBayes v. 3.2.7 (Ronquist et al., 2012) and maximum likelihood (ML) analysis performed with RAxML v.8.2.10 (Stamatakis, 2014) as explained in Yadav et al. (2022, 2023). The phylogenetic analyses were individually applied to two datasets as different combinations were used as barcodes and could provide valuable information for understanding evolutionary relationships at the genus and species level in *Mycosphaerellaceae* (Videira et al., 2017; Chen et al., 2022). Dataset 1 consisted of LSU-*Rpb2* sequences, and dataset 2 consisted of LSU-*Rpb2*-ITS sequences from 32 genera currently known to the *Mycosphaerellaceae*. All trees were rooted with *Ramichloridium apiculatum* (CBS 156.59) and *Uwebraunia australiensis* (CBS 120729).

**Figure 1:**
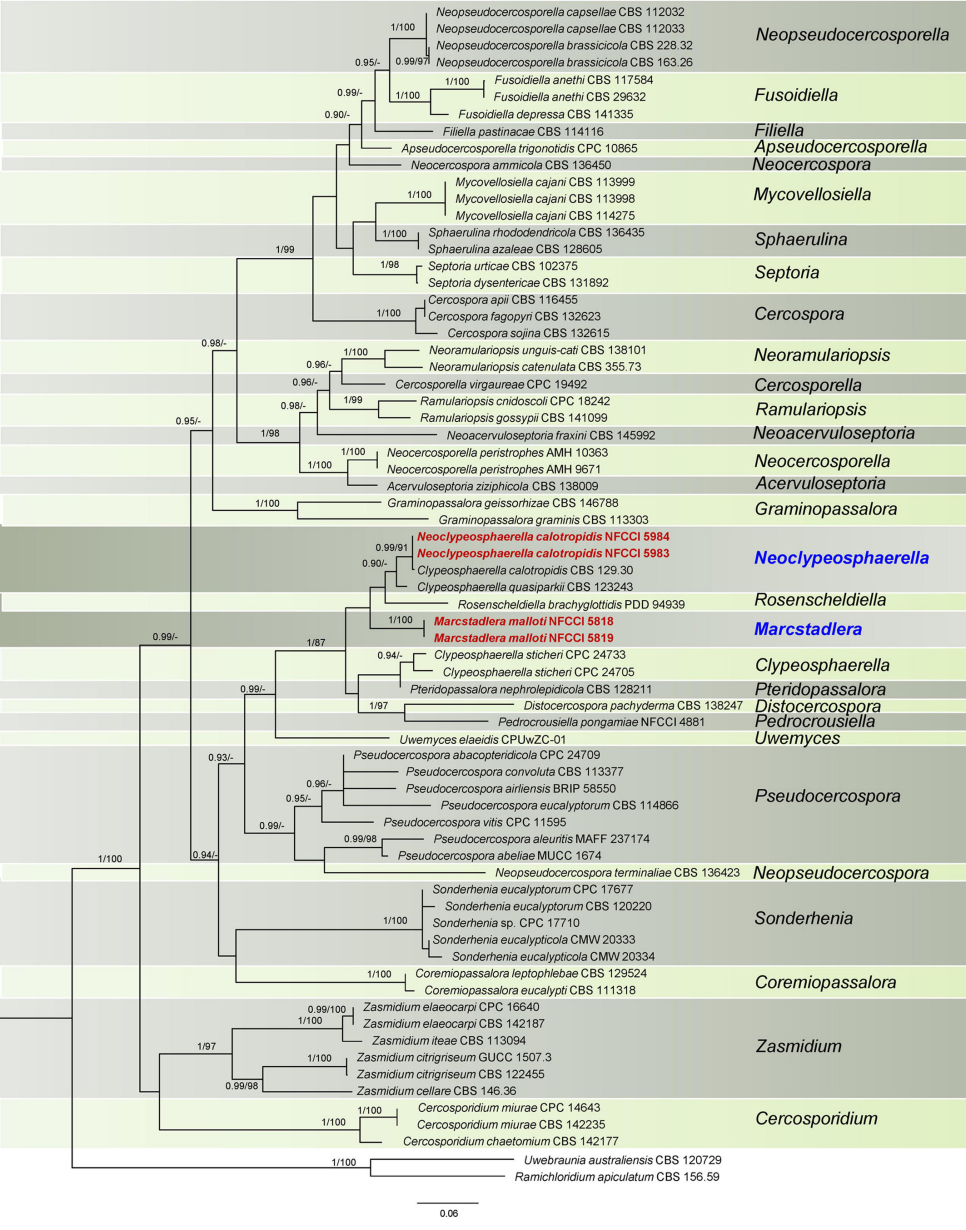
Phylogenetic tree resulting from a RAxML analysis of the combined LSU-*Rpb2* sequence alignment (dataset 1). The Bayesian posterior probabilities (≥0.90; BI-PP) and maximum likelihood bootstrap support values (≥85%; ML-BS) are given at the nodes (BI-PP/ML-BS). The newly introduced lineage is represented in red bold and novel genera denoted in blue. The tree is rooted to *Ramichloridium apiculatum* CBS 156.59 and *Uwebraunia australiensis* CBS 120729.

**Figure 2:**
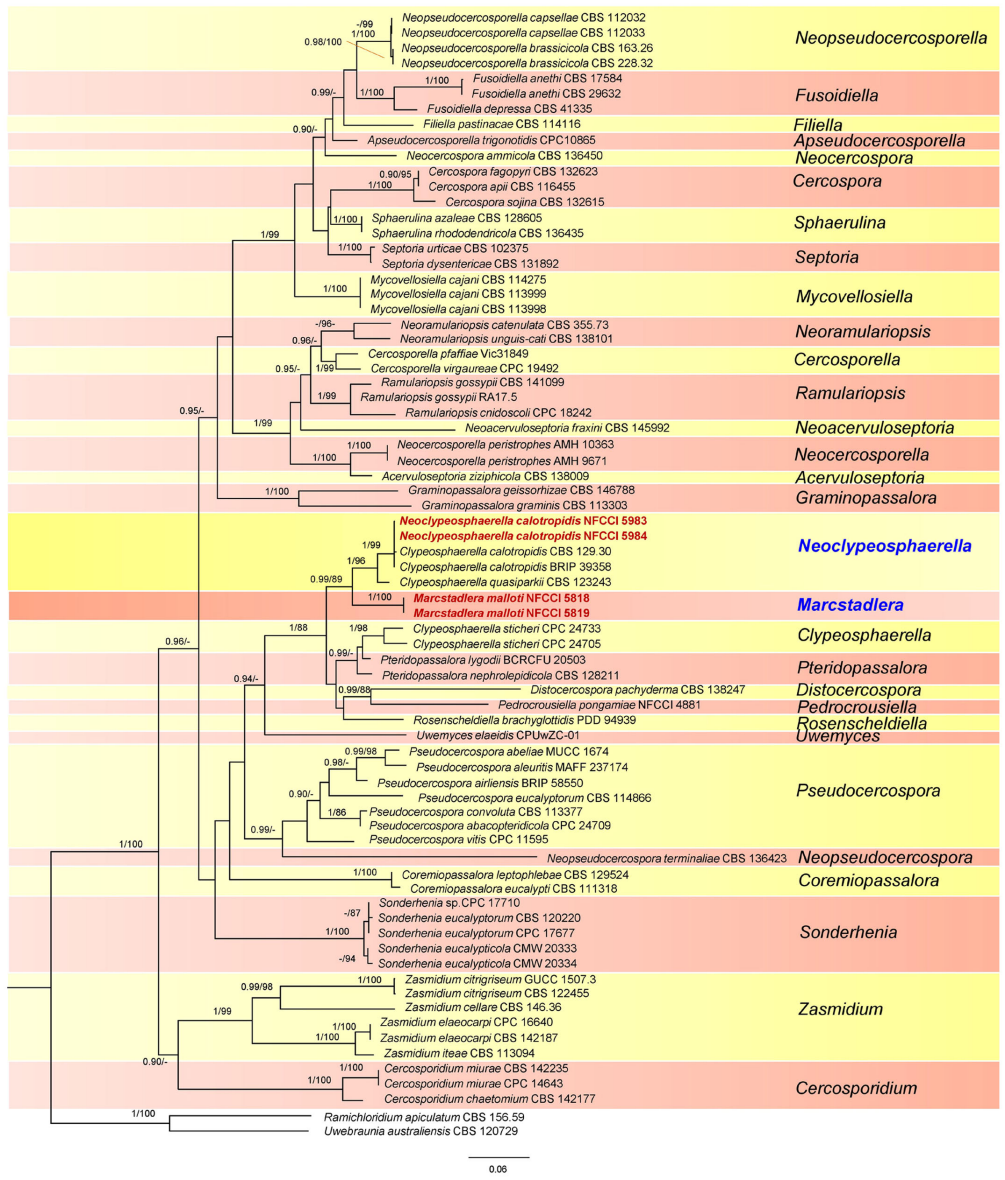
Phylogenetic tree resulting from a RAxML analysis of the combined LSU-*Rpb2*-ITS sequence alignment (dataset 2). The Bayesian posterior probabilities (≥0.90; BI-PP) and maximum likelihood bootstrap support values (≥85%; ML-BS) are given at the nodes (BI-PP/ML-BS). The newly introduced lineage is represented in red bold and novel genera denoted in blue. The tree is rooted to *Ramichloridium apiculatum* CBS 156.59 and *Uwebraunia australiensis* CBS 120729.

The phylogenetic trees of ML were visualized using FigTree v.1.4.4 and edited using Adobe^®^ Illustrator v. CC 2017 (**Figures 1**, **2**).

### Genealogical concordance phylogenetic species recognition analysis

**Figure 3:**
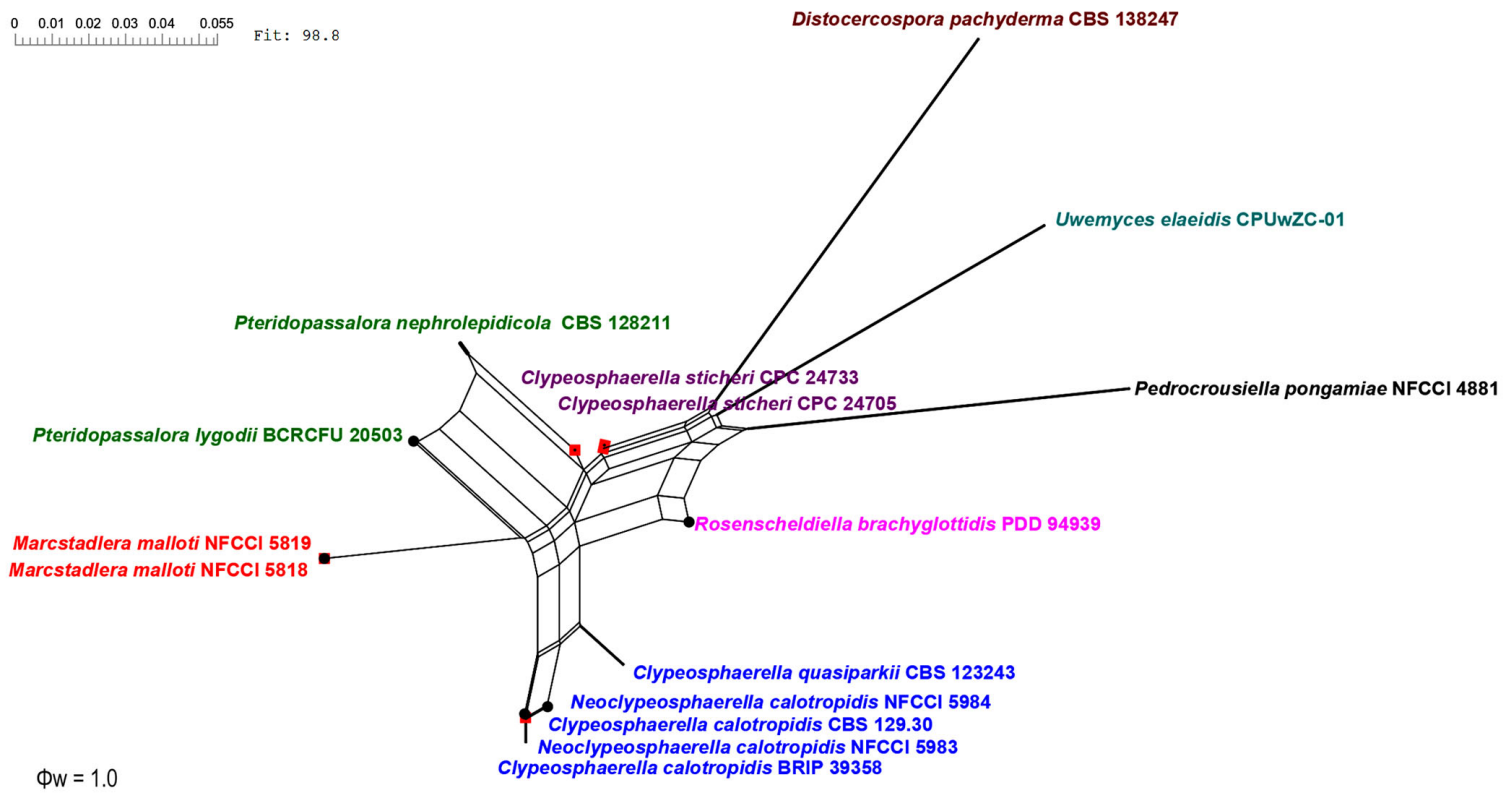
Split graphs showing the results of the pairwise homoplasy index (PHI) test of closely related species using both LogDet transformation and splits decomposition. PHI test results (Φw) ≤0.05 from the PHI test denotes the presence of significant recombination within the dataset. The newly identified taxa are shown in red and blue.

The genealogical concordance phylogenetic species recognition (GCPSR) model (as described by Taylor et al., 2000) was used to clarify species boundaries among closely related and potentially ambiguous taxa by using a pairwise homoplasy index (Φw) test, a statistical test to evaluate genetic data. GCPSR is valued for its ability to synthesize information from multiple genes, evaluate gene flow, operate within an evolutionary timescale, and provide practical insights into species delimitation. It underscores the complexity of species boundaries and offers a robust framework for understanding evolutionary relationships among organisms (Koufopanou et al., 1997; Geiser et al., 1998; Taylor et al., 2000; Starkey et al., 2007). A pairwise homoplasy index (PHI) test (Philippe and Bryant, 2006) was performed in SplitsTree4 (Huson, 1998; Huson and Bryant, 2006) to determine the recombination level within phylogenetically closely related species using a three-locus concatenated dataset of closely related species. If the PHI value exceeds the threshold of 0.05 (Φw ≥ 0.05), it signifies the absence of significant recombination in the dataset. The relationships between these 15, closely related, species were visualized by constructing split graphs (**Figure 3**) from the three-locus concatenated datasets, using both the Log-Det transformation and splits decomposition options.

## Results

The sequences from specimens NFCCI 5818 and NFCCI 5819 were 100% identical across all regions. Likewise, the sequences from specimens NFCCI 5983 and NFCCI 5984 were also 100% identical in each region. The data for the trees conducted in the different analyses are shown in **Table 1**. Phylogenetic trees obtained from the combined gene analyses are supplied below (**Figures 1**, **2**).

### Dataset 1 (LSU-*Rpb2 *phylogeny)

This dataset consisted of a concatenated alignment of two loci: LSU and *Rpb2*. The final alignment has a total of 1,235 characters, with LSU contributing 692 characters and *Rpb2* contributing 543 characters, inclusive of alignment gaps. The phylogenetic trees generated from Bayesian analyses (BI) and maximum parsimony (MP) have shown similar overall topology, indicating consistent results across these methods (Videira et al., 2017). The best scoring RAxML tree is presented in **Figure 1**, with the likelihood value of −14,653.366735. Estimated base frequencies were as follows: A = 0.235837, C = 0.305805, G = 0.252457, T = 0.205901; substitution rates AC = 1.073482, AG = 3.362976, AT = 0.756117, CG = 0.718572, CT = 5.587844, GT = 1.000000; gamma distribution shape parameter *α* = 0.547561. In this analysis, *Clypeosphaerella calotropidis* (CBS 129.30) and *C. quasiparkii* (CBS 123243) are now separated from the *Clypeosphaerella* (type species: *C. sticheri*) clade and are placed in a separate sister branch of *Rosenscheldiella brachyglottidis* (PDD 94939) along with NFCCI 5983 and NFCCI 5984 (**Figure 1**). *Clypeosphaerella calotropidis*, *C. quasiparkii*, and *C. sticheri* form a paraphyletic group. *Marcstadlera* is identified as a sister group to both *Neoclypeosphaerella* and *Rosenscheldiella.* However, the statistical support for this relationship is very low (BI-PP/ML-BS: 0.51/37).

### Dataset 2 (LSU-*Rpb2*-ITS phylogeny)

This dataset consisted of a concatenated alignment of three loci: LSU, *Rpb2*, and ITS. The final alignment of this dataset contained a total of 1,675 characters divided into three partitions containing 692 (LSU), 543 (*Rpb2*), and 440 (ITS) characters, including alignment gaps. The phylogenetic trees generated from BI and MP have shown similar overall topology, indicating consistent results across these methods. The best scoring RAxML tree is presented in **Figure 2**, with the likelihood value of −19,399.308522. Estimated base frequencies were as follows: A = 0.201081, C = 0.284874, G = 0.278655, T = 0.235390; substitution rates AC = 1.857707, AG = 4.458699, AT = 1.107011, CG = 0.007891, CT = 6.730028, GT = 1.000000; gamma distribution shape parameter *α* = 0.484710. The results of the analysis of dataset 2 (**Figure 2**) strongly supported the dataset 1 analysis, except for the placement of *Rosenscheldiella brachyglottidis* (PDD 94939) (**Figure 1**). *Marcstadlera* is identified as a sister group to *Neoclypeosphaerella* with high statistical support (BI-PP/ML-BS: 0.99/89), suggesting a close evolutionary relationship between these two genera.

In both datasets, *C. calotropidis* (BRIP 39358 and CBS 129.30) and *C. quasiparkii* (CBS 123243) are separately clustered from the type species of *Clypeosphaerella*, *C. sticheri* (CPC 24705 and CPC 24733), and form a paraphyletic group. Both *C. calotropidis* and *C. quasiparkii* are grouped with NFCCI 5983 and NFCCI 5984 (*Neoclypeosphaerella calotropidis*) in a distinct sister branch of the newly introduced genus *Marcstadlera* (**Figure 2**), forming a statistically supported monophyletic group (BI-PP/ML-BS: 0.99/89).

*Clypeosphaerella*, *Distocercospora*, *Marcstadlera*, *Neoclypeosphaerella*, *Pedrocrousiella*, *Pteridopassalora*, and *Rosenscheldiella* form a statistically supported monophyletic group in both datasets (BI-PP/ML-BS: 1/87 or 1/88).

### Genealogical concordance phylogenetic species recognition analysis

The PHI tests were carried out to calculate the recombination level within two novel genera and their phylogenetically closely related taxa. The PHI tests showed that there was no significant recombination (Фw = 1.0) between closely related taxa, viz., *Clypeosphaerella*, *Distocercospora*, *Marcstadlera*, *Neoclypeosphaerella*, *Pedrocrousiella*, *Pteridopassalora*, *Rosenscheldiella*, and *Uwemyces* (**Figure 3**).

### Taxonomy

**Figure 4:**
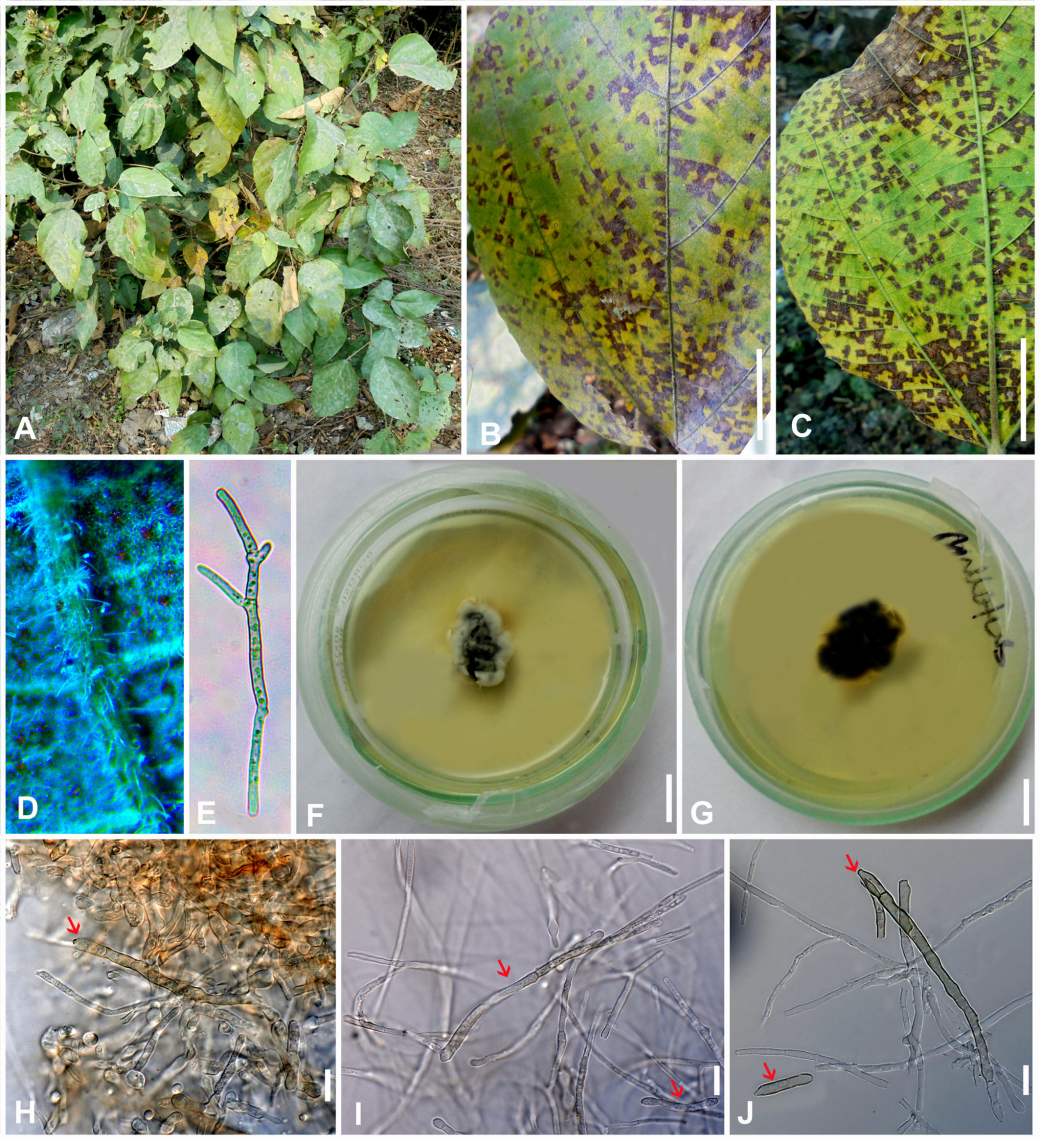
*Marcstadlera malloti* (AMH 10726) on *Mallotus philippensis* (*Euphorbiaceae*). **(A)** *Mallotus philippensis* in natural habitat. **(B)** Symptoms on upper leaf surface. **(C)** Symptom on lower leaf surface. **(D)** Close-up of leaf surface showing fungal fructifications. **(E)** Germinating conidium. **(F)** Top view of ex-epitype culture on PDA. **(G)** Reverse view of ex-epitype culture on PDA. **(H–J)** Mycelia from ex-epitype culture showing formation of chlamydospores and conidia (showing arrows for conidia). Scale bars: **(B, C, F, G)** 20 mm, **(H–J)** 10 µm.

**Figure 5:**
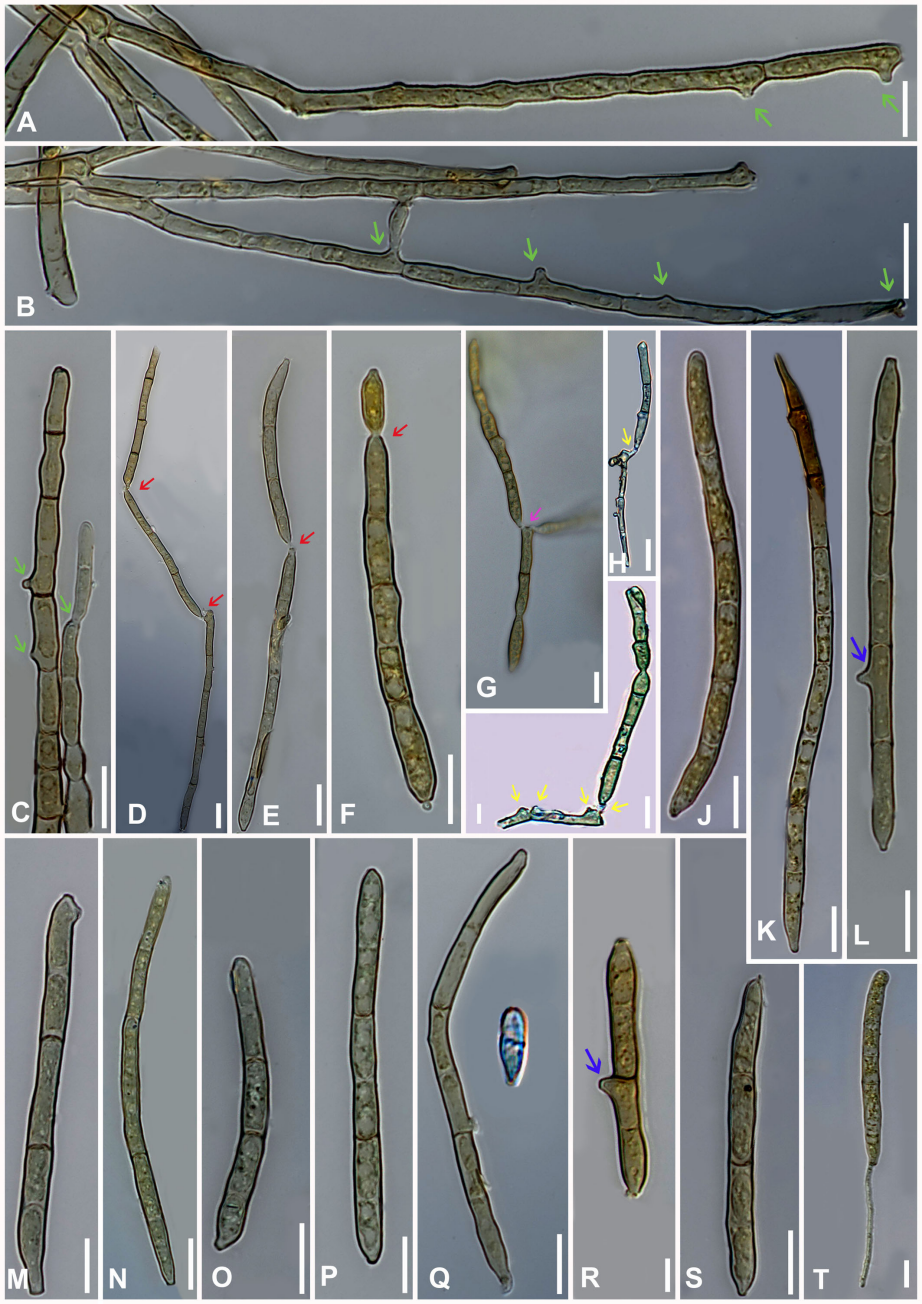
Microphotographs of *Marcstadlera malloti* (AMH 10726). **(A–C)** Superficial hyphae with developing conidiogenous cells (green arrows for conidiogenous loci). **(D–F)** Conidia in chain (red arrows for catenation). **(G)** Ramoconidia with branched catenation (pink arrow for branched catenation). **(H, I)** Superficial hyphae with conidiogenous loci bearing conidia (yellow arrows for conidiogenous loci). **(J–S)** Conidia (blue arrows for the development of conidiogenous loci). **(T)** Monopolar germination in conidium. Bars: 10 µm.

**Figure 6:**
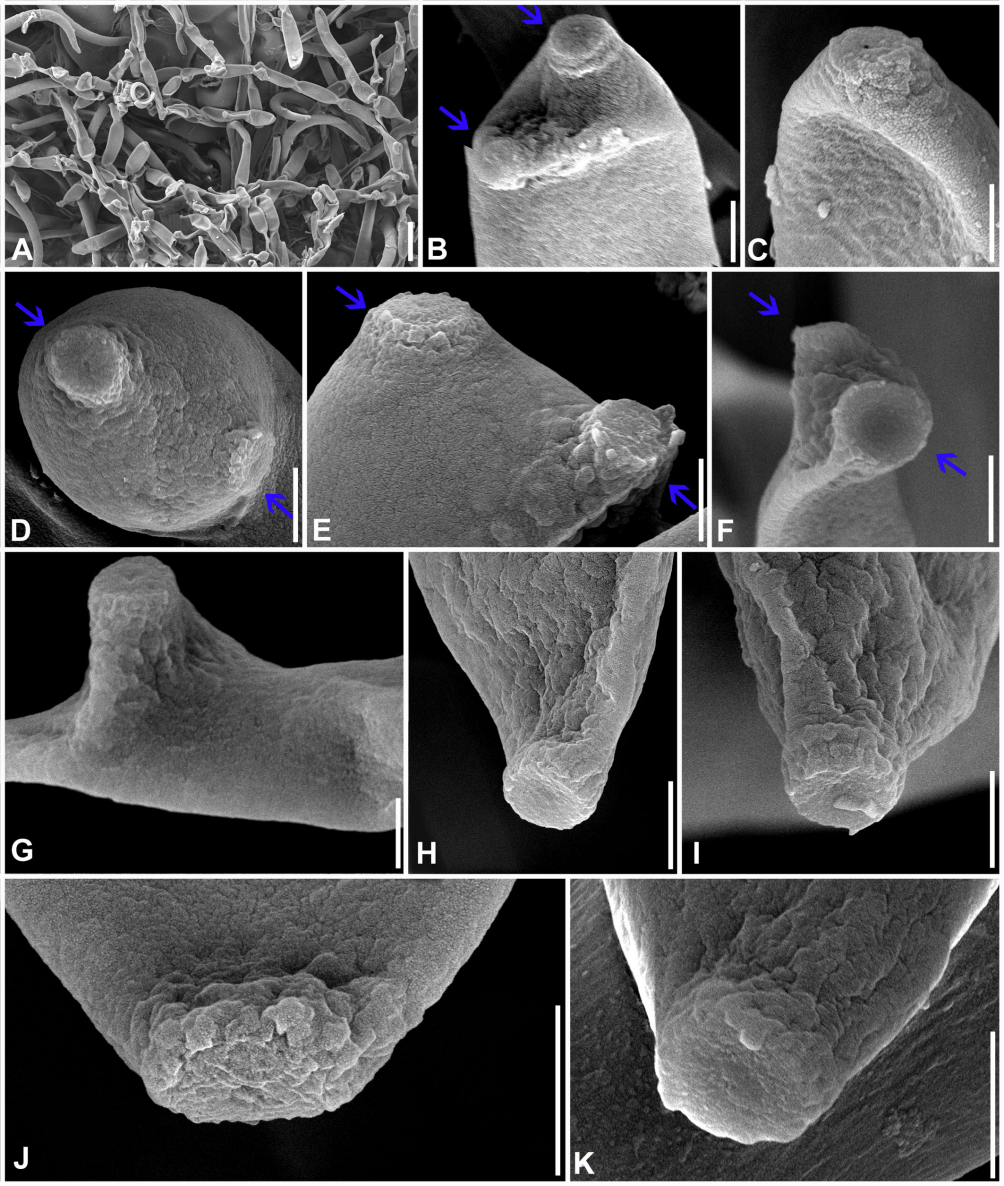
Scanning electron microphotographs of *Marcstadlera malloti* (AMH 10726). **(A)** Superficial hyphae with conidia and developing conidiogenous cells. **(B–G)** Top and lateral views of conidiogenous loci (blue arrows for polyblastic nature of conidiogenous cells). **(H–K)** Top and lateral view of hila of conidia. Scale bars: **(A)** 10 µm, **(B–K)** 1 µm.

***Marcstadlera*** Gargee Singh & Raghv. Singh, **gen. nov.** (**Figures 4**–**6**).

MycoBank number: MB854795.

Etymology: This is derived from the name of Professor Dr. Marc Stadler (Helmholtz Centre for Infection Research, Braunschweig, Germany), a globally renowned expert in industrial microbiology and mycology, as well as fungal biodiversity research and natural product chemistry.

Diagnosis: This differs from the genus *Neoclypeosphaerella* by developing conidiophores reduced to conidiogenous cells, arising singly from external hyphae as intercalary or terminal cells of superficial hyphae, micronematous to semi-macronematous, mononematous, unbranched, aseptate, and mostly catenate conidia.

Description: Phytopathogenic, causing leaf spots. *Stromata* absent. *Mycelium* mostly external and superficial, septate, branched, smooth to slightly roughened, light brown or olivaceous brown. *Conidiophores* micronematous, reduced to conidiogenous cells or semi-macronematous, mononematous, developing individually from intercalary or terminal cells of external hyphae, unbranched, aseptate, light brown or olivaceous brown. *Conidiogenous cells* mono- to polyblastic, cylindrical or peg-like, integrated, conidiogenous loci (scars) flattened (ultrastructure), unthickened, to slightly thickened and darkened. *Ramoconidia* obclavate-cylindrical, smooth to slightly roughened, light brown to pale olivaceous brown. *Conidia* blastocatenate, obclavate-cylindrical, transversely septate, straight to curved, thick-walled, tip subacute to rounded, base narrowly obconical, truncate (ultrastructure), smooth to slightly roughened, pale brown to pale olivaceous-brown, hilum unthickened to slightly thickened and darkened.

Type species: *Marcstadlera malloti* (Kharwar, P.N. Singh and R.K. Chaudhary) Gargee Singh, Raghv. Singh, and Sahana.

Notes: Based on a megablast search of NCBI’s GenBank nucleotide database, the closest hits using the ITS sequence had the highest similarity to *Clypeosphaerella quasiparkii* [strain CBS 123243, GenBank MH863287; identities = 416/434 (96%), 3 gaps (0%)], *Clypeosphaerella calotropidis* [strain BRIP 39358, GenBank AY303969; identities = 402/418 (96%), 2 gaps (0%)], and *Ramulariopsis gossypii* [strain RA17.5, GenBank KR265337; identities = 417/441 (95%), 14 gaps (3%)]. Closest hits using the LSU sequence are *Clypeosphaerella sticheri* [strain CPC 24733, GenBank KT037577; identities = 516/527 (98%), 0 gap (0%)], *Pteridopassalora nephrolepidicola* [strain CBS 128211, GenBank HQ599591; identities = 516/527 (98%), 0 gap (0%)], and *Clypeosphaerella quasiparkii* [strain CBS 123243, GenBank MH874811; identities = 516/529 (98%), 2 gap (0%)]. Closest hits using the *Rpb2* sequence had the highest similarity to *Clypeosphaerella calotropidis* [strain CBS 129.30, GenBank MF951477; identities = 517/586 (88%), 0 gaps (0%)], *Clypeosphaerella quasiparkii* [strain CBS 123243, GenBank MF951478; identities = 515/586 (88%), 0 gaps (0%)], and *Pteridopassalora nephrolepidicola* [strain CBS 128211, GenBank KX462646; identities = 492/576 (85%), 0 gaps (0%)].

***Marcstadlera malloti*** (Kharwar, P.N. Singh & R.K. Chaudhary) Gargee Singh, Raghv. Singh, & Sahana **comb. nov.** (**Figures 4**-**6**).

MycoBank number: MB854796.

≡ *Mycovellosiella malloti* Kharwar, P.N. Singh and R.K. Chaudhary, *Mycol. Res.* 100(6), 689 (1996).

= *Pseudocercospora malloti* (Kharwar, P.N. Singh and R.K. Chaudhary) U. Braun, *Schlechtendalia* 19, 69 (2009).

Description: *Leaf* sp*ots* amphiphyllous, angular, grayish brown to dark brown, vein-limited, 1–2.5 mm wide, sometimes coalescing. *Colonies* effuse, hypogenous, grayish brown, velvety. *Stromata* absent. *Mycelium* mostly external and superficial, septate, branched, smooth to slightly roughened, light brown or olivaceous brown, 2–4.5 μm wide. *Conidiophores* micronematous, reduced to conidiogenous cells or semi-macronematous, mononematous, developing individually from intercalary or terminal cells of external hyphae, unbranched, aseptate, light brown or olivaceous brown, (10–)12–15(–20) × (2–)2.5–3(–3.5) μm. *Conidiogenous cells* mono to polyblastic, determinate or sympodial elongated, cylindrical or peg-like, integrated, 1.5–3 × 1–1.5 μm. *Conidiogenous loci* (scars) complanate, unthickened to slightly thickened and darkened, flattened at the apex. *Ramoconidia* intercalary, obclavate-cylindrical, smooth to slightly roughened, light brown to pale olivaceous brown. *Conidia* blastocatenate, obclavate-cylindrical, apex subacute to rounded, base narrowly obconical truncate, straight to curved, 1–8-euseptate, smooth to slightly roughened, pale brown to pale olivaceous brown, dry, (10–)45–78(–117) × (2.5–)3–4(–5.5) μm, thick-walled, hilum unthickened to slightly thickened and darkened, 0.8–1.5 μm wide.

Culture characteristics: Colonies on PDA slow-growing and attained a diameter of approximately 30 mm after 21 days at 25 °C ± 5 °C, raised, irregular, aerial mycelium velvety, upper surface dark gray to black centrally and white fluffy at the periphery, reverse brown to black. Cultures fertile. *Hyphae* 1.5–2.5 μm wide, branched, septate, smooth to slightly roughened, subhyaline to very light olivaceous brown. *Conidiophores* micronematous, reduced to conidiogenous cells or semi-macronematous, mononematous, unbranched, aseptate, hyaline to very light olivaceous brown, (12–)20–22(–25) × (2–)2.5–3(–3.5) μm. *Conidiogenous cells* monoblastic, cylindrical or peg-like, intercalary and terminal, determinate. *Conidiogenous loci* (scars) unthickened to slightly thickened and darkened, loci cylindrical or peg-like, 1.5–3 × 1–1.5 μm. *Conidia* solitary, obclavate-cylindrical, base narrowly obconical truncate, apex subacute to rounded, straight to curved, 1–8-euseptate, smooth to slightly roughened, light brown to pale olivaceous brown, (25–)70–100(–110) × (3–)4–4.5(–5) μm, hilum unthickened to slightly thickened and darkened, 1–1.5 μm wide. *Chlamydospores* spherical to oval, light brown to mid brown, smooth, 2–7 × 2–5 μm.

Specimens examined: NEPAL, Chitwan, Narayangarh, on living leaves of *Mallotus philippensis* (Lam.) Müll. Arg. (*Euphorbiaceae*), January 1995, Kamal (GPU 3008, HCIO 41505 isotype, IMI 366204 holotype); INDIA, Uttar Pradesh, Gorakhpur, Kushmi Forest, 26.749748°N 83.468645°E, on living leaves of *Mallotus philippensis*, 8 February 2023, Gargee Singh, MH-BHU 114 (AMH 10726, epitype designated here, MycoBank MBT10024805), ex-type culture NFCCI 5818, gene sequence GenBank: PQ012587 (ITS), PQ012588 (LSU), PQ034553 (*Rpb2*); INDIA, Uttar Pradesh, Gorakhpur, Kushmi Forest, on living leaves of *M. philippensis*, 25 March 2024, Raghvendra Singh, MH-BHU 115(AMH 10727), culture NFCCI 5819, gene sequence GenBank: PQ013688 (ITS), PQ013689 (LSU), PQ034554 (*Rpb2*).

Note: Currently, five species of *Pseudocercospora* have been described on *Mallotus*, namely, *P. bakeriana* Deighton [≡ *Cercospora bakeriana* Saccardo 1914] (Deighton, 1976), *P. malloti* (Kharwar, P.N. Singh and R.K. Chaudhary) U. Braun [≡ *Mycovellosiella malloti* Kharwar, P.N. Singh and R.K. Chaudhary] (Kharwar et al., 1996; Braun, 2009), *P. malloti-repandi* (Bhalla, S.K. Singh and A.K. Srivast.) U. Braun [≡ *Mycovellosiella malloti-repandi* Bhalla, S.K. Singh and A.K. Srivast.] (Bhalla et al., 1997; Braun, 2000b), *P. melanolepidis* Goh and W.H. Hsieh (Goh and Hsieh, 1987), and *P. pampangensis* (Petr.) U. Braun [≡ *Cercospora pampangensis* Petr.] (Petrak, 1956; Braun, 1996).

In comparison to *Marcstadlera malloti*, *P. bakeriana* exhibits several distinctive features in its development of conidiophores. Notably, *P. bakeriana* forms fascicles of conidiophores that arise from both external and internal hyphae. These conidiophores can range from simple to highly branched ones. They are septate and can reach lengths of up to 130 μm with widths varying between 3 and 6 μm. This morphological variability in conidiophore structure is a significant distinguishing characteristic between the two species.

*Marcstadlera malloti* appears to be most closely related to *P. malloti* and *P. malloti-repandi.* Both species develop superficial hyphae that give rise to micronematous to semi-macronematous, mononematous conidiophores, either terminally or as lateral branches. The solitary to catenate nature of conidia in *P. malloti* closely resembles those of *M. malloti*, making them morphologically indistinguishable. As a result, *P. malloti* is used as the type species for *Marcstadlera*. In contrast, *P. malloti-repandi* can be differentiated by its branched, septate, and longer, wider conidiophores (2.5–65 × 2.5–6 μm).

In addition to the formation of stromata and fascicles of primary conidiophores (20–65 × 3–4 μm), *P. melanolepidis* develops secondary external hyphae that bear secondary conidiophores (up to 10 μm long) both terminally and laterally. Notably, both types of conidiophores are septate.

*Pseudocercospora pampangensis* develops stromata that bear large clusters (fascicles) of subsynnematous conidiophores. These conidiophores are occasionally branched, pluriseptate, and relatively larger in size (15–250 × 3–6 μm).

**Figure 7:**
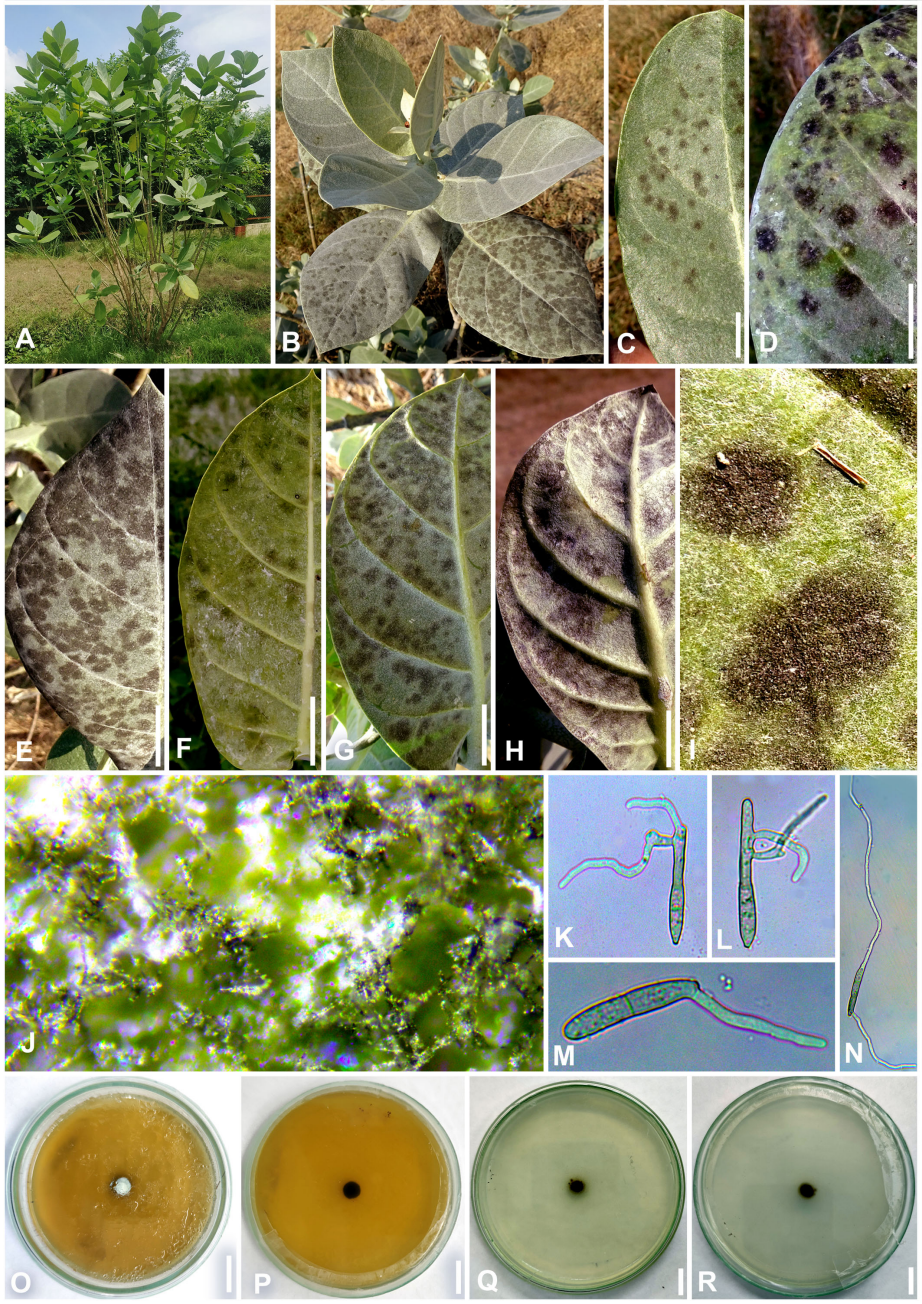
*Neoclypeosphaerella calotropidis* (AMH 10781) on *Calotropis gigantea* (*Apocynaceae*). **(A, B)** *Calotropis gigantea* in natural habitat. **(C–E)** Symptoms on the upper surfaces of leaves. **(F–H)** Symptom on the lower surfaces of leaves. **(I)** Stereoscopic view of infection spots. **(J)** Close-up of leaf symptoms showing fungal fructifications. **(K–N)** Germinating conidia. **(O)** Top view of ex-epitype culture on MEA. **(P)** Reverse view of ex-epitype culture on MEA. **(Q)** Top view of ex-epitype culture on PDA. **(R)** Reverse view of ex-epitype culture on PDA. Scale bars: **(C–H)** 20 mm, **(O–R)** 10 mm.

**Figure 8:**
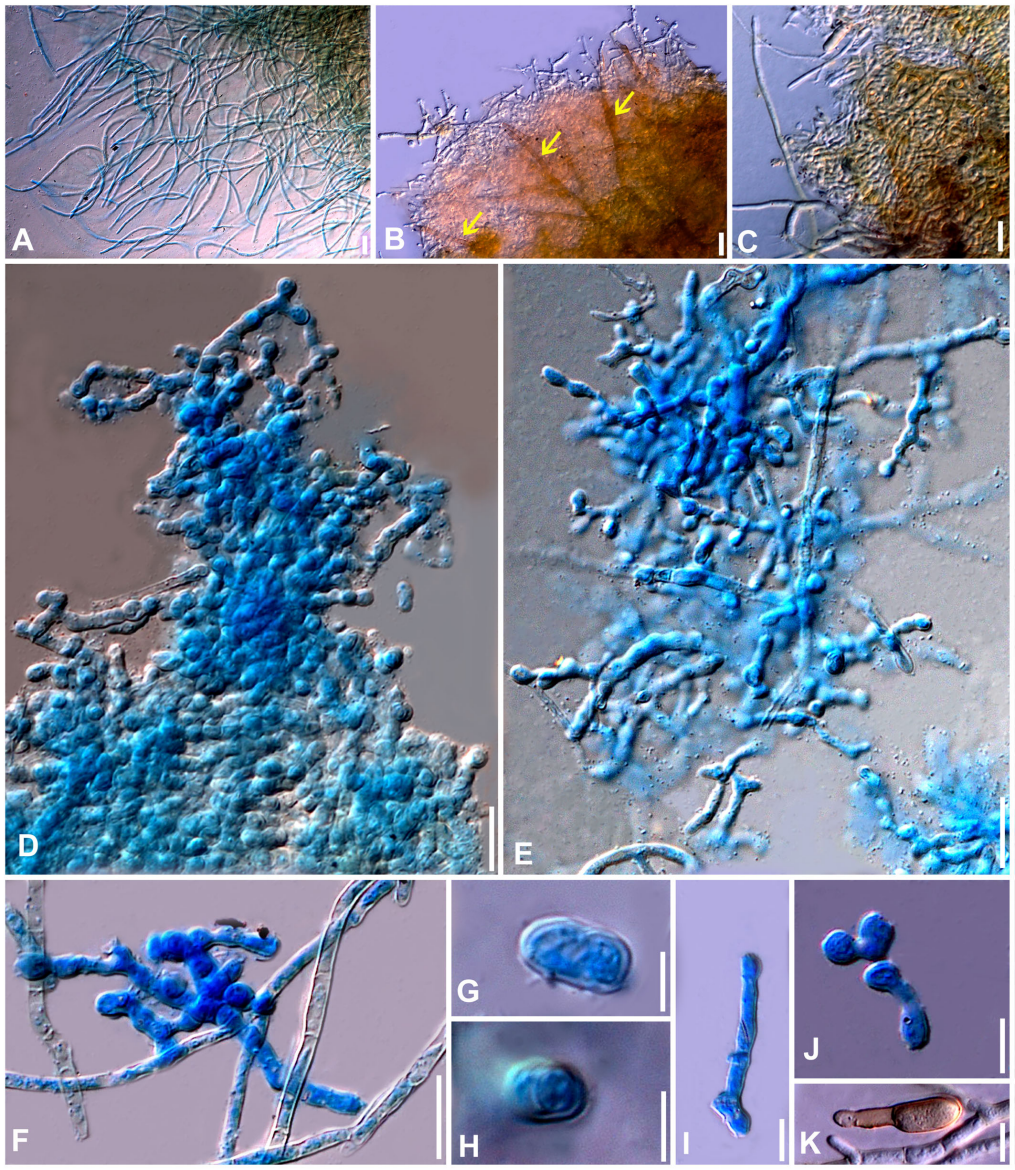
Microphotographs of the ex-type culture of *Neoclypeosphaerella calotropidis* (NFCCI 5983) on MEA. **(A)** Hyphae. **(B, C)** Fructification showing formation of chlamydospores with setae-like structures (yellow arrows for setae-like structures). **(D–F)** Developing chains of intercalary and terminal chlamydospores. **(G, H)** Chlamydospores. **(I–K)** Germinating chlamydospores. Scale bars: **(A–F)** 20 µm, **(G, H)** 5 µm, **(I–K)** 10 µm.

**Figure 9:**
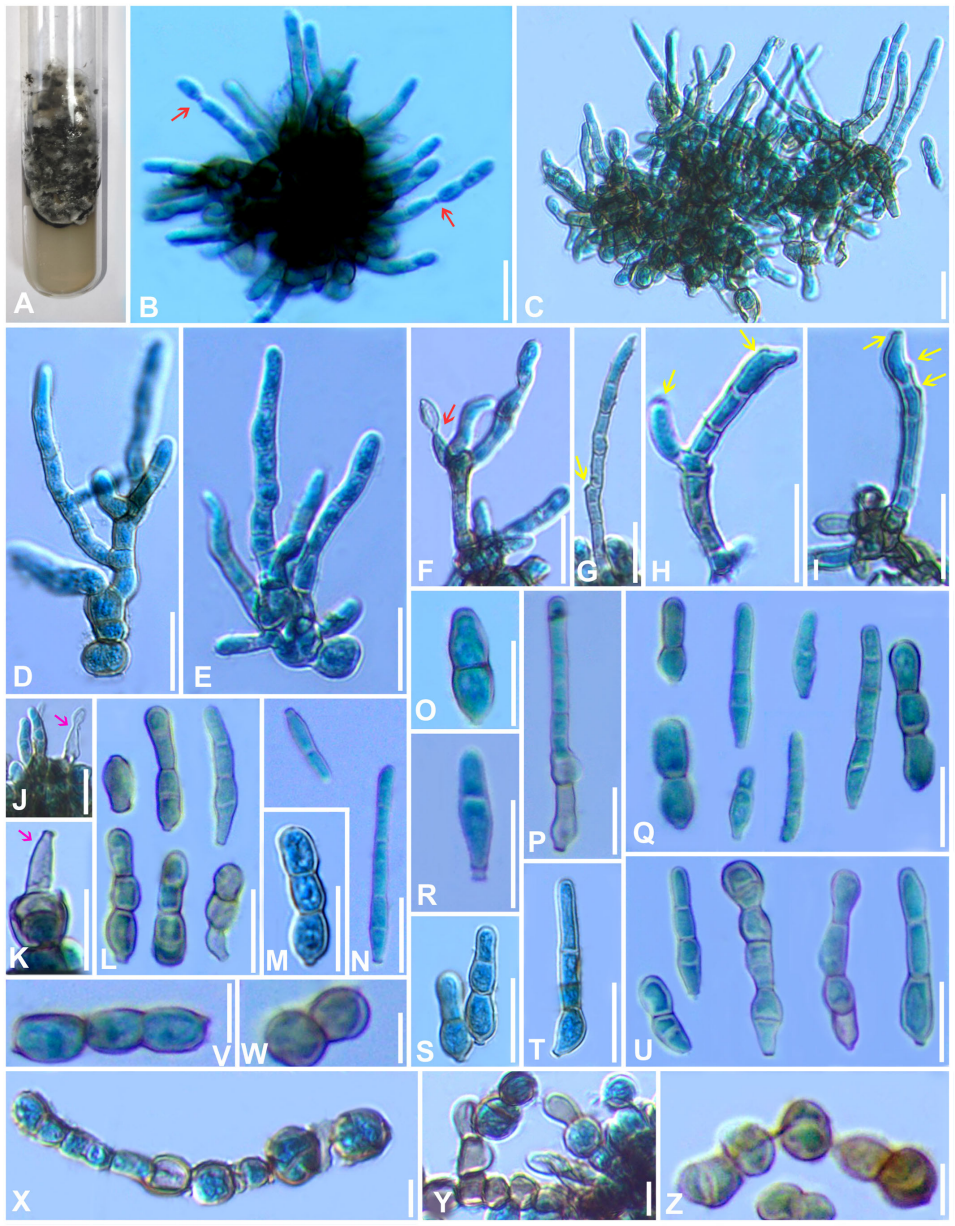
Microphotographs of the ex-type culture of *Neoclypeosphaerella calotropidis* (NFCCI 5983) on agar media supplemented with undefined vegetable peelings. **(A)** Top view of ex-epitype culture. **(B, C)** Stromata with fascicles of conidiophores (red arrows for developing conidia). **(D–F)** Highly branched conidiophores. **(G–I**) Conidiogenous cells with loci (yellow arrows). **(J, K)** Ampulliform conidiogenous cells (pink arrows). **(L–U)** Conidia. **(V–Z)** Chlamydospores in chain. Scale bars: **(B–T)** 20 µm, **(U–Z)** 10 µm.

**Figure 10:**
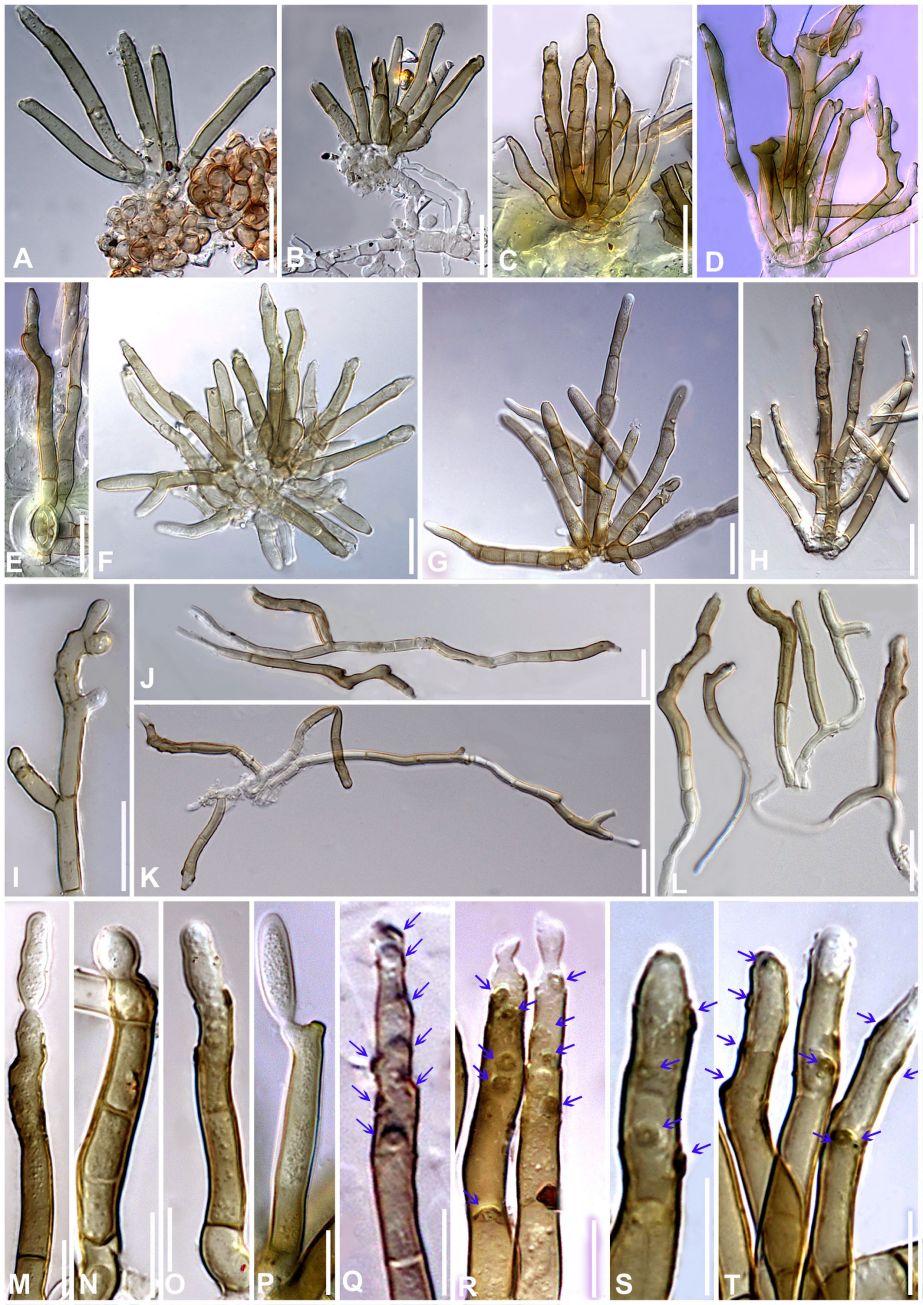
Microphotographs of *Neoclypeosphaerella calotropidis* (AMH 10781). **(A)** Stromata with fascicles of conidiophores. **(B)** Stromata with fascicles of conidiophores and superficial hyphae. **(C–E)** Fascicles of conidiophores emerges through stomata. **(F, G)** Erumpent stromata bearing conidiophores with swollen basal cell. **(H, I)** Highly branched conidiophores. **(J–L)** Superficial hyphae with conidiophores. **(M–P)** Conidiophores with developing conidia. **(Q–T)** Polyblastic nature of conidiogenous cells (blue arrows). Scale bars: **(A–L)** 20 µm, **(M–T)** 10 µm.

**Figure 11:**
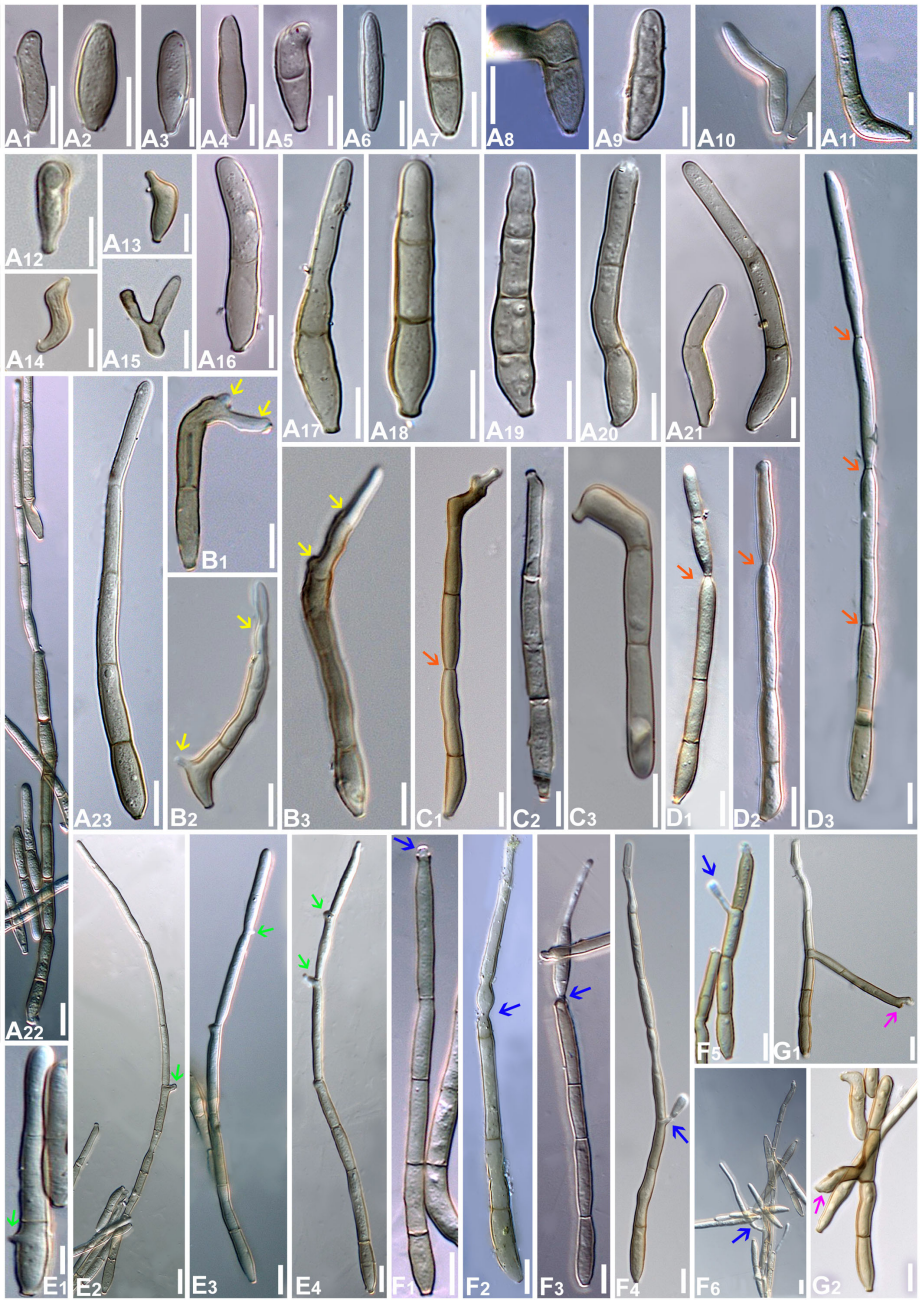
Microphotographs of *Neoclypeosphaerella calotropidis* (AMH 10781). **(A1–A23)** Conidia. **(B1–B3)** Ramoconidia with apical hila and developing conidium (yellow arrows). **(C1–C3)** Intercalary conidia. **(D1–D3)** Conidia in catenation (orange arrows for point of catenation). **(E1–E4)** Conidiogenous nature of conidial cells (green arrows). **(F1–F6)** Conidia with developing conidium (blue arrows). **(G1, G2)** Development of conidiophores from conidial cells (pink arrows). Bars: 10 µm.

**Figure 12:**
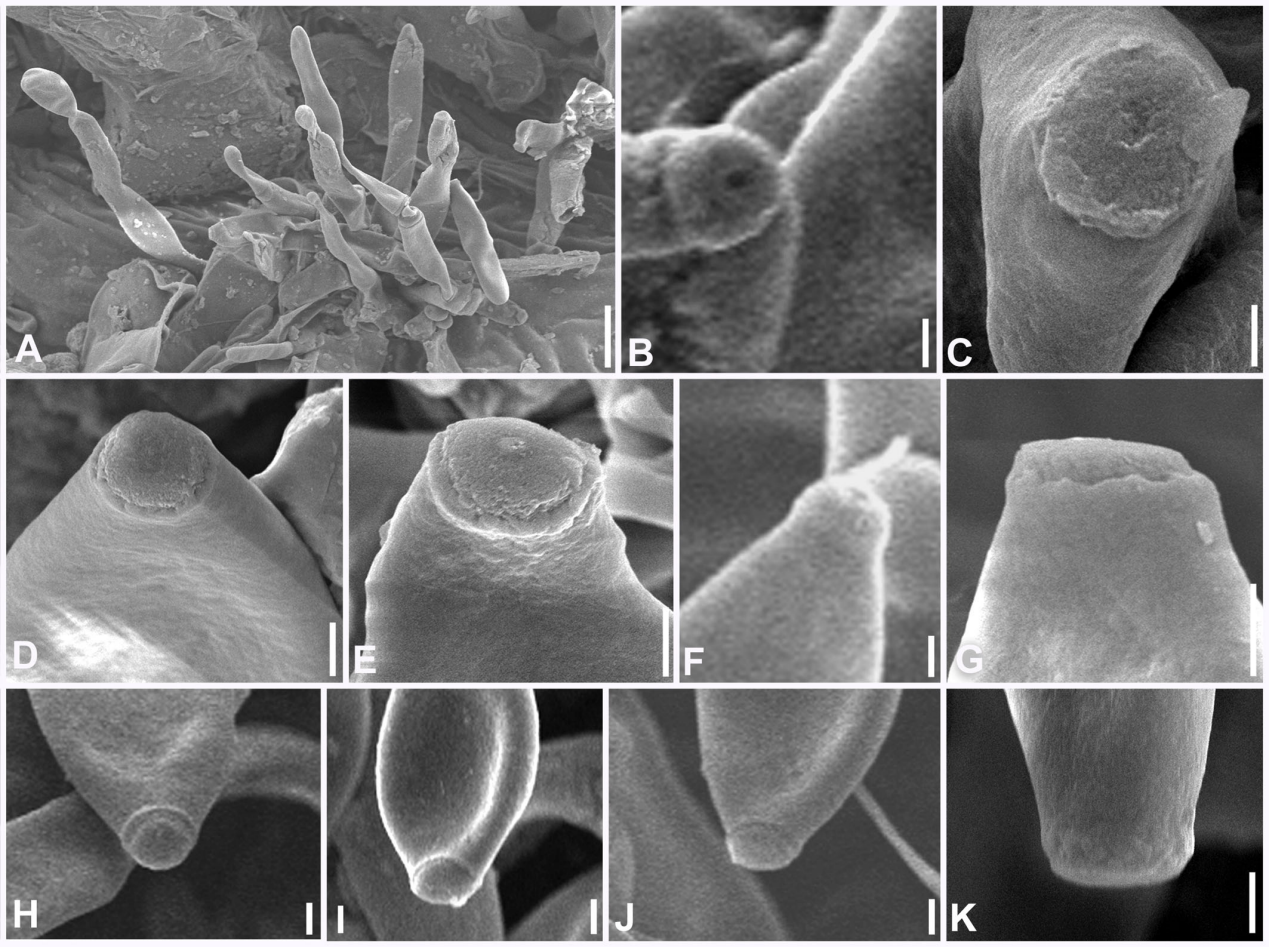
Scanning electron microphotographs of *Neoclypeosphaerella calotropidis* (AMH 10781). **(A)** Fascicle of conidiophores with conidia. **(B–G)** Top and lateral view of loci of conidiogenous cells. **(H–K)** Top and lateral view of hila of conidia. Scale bars: **(A)** 10 µm, **(B–K)** 1 µm.

***Neoclypeosphaerella*** S. Rajwar & Raghv. Singh, **gen. nov.** (**Figures 7**–**12**).

MycoBank number: MB854797.

Etymology: This is composed of Neo- (new) and the genus name *Clypeosphaerella*.

Diagnosis: This differs from the genus *Marcstadlera* by developing fascicles of conidiophores emerging from stromata and conidia that are rarely catenate.

Description: Plant pathogenic. *Ascomata* epiphyllous, black, subepidermal to erumpent, subglobose, wall of 3–4 layers of medium to dark brown *textura angularis*, apical ostiole central. *Asci* aparaphysate, fasciculate, bitunicate, subsessile, broad ellipsoid to obclavate, straight to slightly curved, 8-spored. *Ascospores* bi- to multiseriate, overlapping, hyaline, guttulate, thin-walled, straight to slightly curved, ellipsoidal to obovoid with obtuse ends, widest in the middle of the apical cell, 1-septate, not constricted at the septum, tapering toward both ends, with a thin mucilaginous sheath. *Conidiophores* macronematous, mostly arising in fascicles from stromata, occasionally as lateral branches of superficial secondary hyphae or conidial cells, erect to slightly curved, divergent, subcylindrical to geniculate-sinuous at the tip, basal cell slightly swollen, mostly unbranched, rarely branched, smooth, sometimes slightly roughened, light brown to brown, septate, thick-walled. *Conidiogenous cells* polyblastic, cylindrical, integrated, terminal and intercalary, sympodial elongated, conidiogenous loci slightly protuberant, surrounded by a circular rim-like structure, forming a truncated apex with a centrally positioned small apical depression, loci thickened and darkened. *Ramoconidia* cylindrical to subcylindrical, rarely sickle-shaped. *Intercalary conidia* sometimes present, cylindrical to subcylindrical, sometimes curved, occurring in chains. *Conidia* sometimes blastocatenate, polymorph, cylindrical to subcylindrical, obclavate-cylindrical, acicular, ovoid to obovoid, doliiform, elliptical, L-shaped to sickle-shaped, rarely V-shaped, obtuse apex, base obconical, truncate at the base, surrounded by a circular rim-like structure, euseptate, smooth to slightly roughened, light olivaceous brown to brown, hilum thickened and darkened.

Type species: *Neoclypeosphaerella calotropidis* (Ellis and Everh.) Raghv. Singh, S. Rajwar, Sanjay, P.N. Singh and U. Braun.

***Neoclypeosphaerella calotropidis*** (Ellis & Everh.) Raghv. Singh, S. Rajwar, Sanjay, P.N. Singh & U. Braun, **comb. nov.** (**Figures 7**-**12**).

MycoBank number: MB854798.

≡ *Cercospora calotropidis* Ellis and Everh., Rep. (Annual) *Missouri Bot. Gard.* 120 (1898).

= *Phaeoramularia calotropidis* (Ellis and Everh.) Kamal, A.S. Moses and R. Chaudhary, *Mycol. Res.* 94, 716 (1990).

= *Passalora calotropidis* (Ellis and Everh.) U. Braun, *Schlechtendalia* 5, 60 (2000).

= *Pseudocercospora calotropidis* (Ellis and Everh.) Haldar and J.B. Ray, *J. Mycopathol. Res.* 39(1), 43 (2001).

= *Clypeosphaerella calotropidis* (Ellis and Everh.) Videira and Crous, *Stud. Mycol.* 87, 314 (2017).

For additional synonyms see Crous and Braun (2003) and MycoBank (https://www.mycobank.org/).

Description: *Leaf* sp*ots* amphiphyllous, initially circular to subcircular, 6–7 mm diam., later irregular and spread over the entire leaf surface, brown to dark blackish brown. *Colonies* amphigenous, effuse, brown to dark brown, velvety. *Mycelium* mostly internal, sometimes superficial secondary hyphae developing from stromata, branched, septate, smooth, thin-walled, hyaline to very light olivaceous, (2−)2.5–3.5(−4) μm. *Stromata* present, globose to subglobose, mostly substomatal, later erumpent, pseudoparenchymatous, light olivaceous brown to mid brown, 20−25 × 15−25 μm. *Conidiophores* macronematous, mostly arising in fascicles from stromata, occasionally as lateral branches of superficial secondary hyphae or conidial cells, erect to slightly curved, divergent, subcylindrical to geniculate-sinuous at the tip, basal cell slightly swollen, mostly unbranched, rarely branched, smooth, sometimes slightly roughened, light brown to brown, 0−8-septate, thick-walled, (17−)25−85(−100) × (3−)3.5−5.5(−6.5) μm. *Conidiogenous cells* integrated, terminal as well as intercalary, polyblastic, cylindrical, conidiogenous loci slightly protuberant, surrounded by a circular rim-like structure, forming a truncated apex with a centrally positioned small apical depression (ultrastructure), loci thickened and darkened, 1.5−2 μm wide. *Ramoconidia* cylindrical to subcylindrical, rarely sickle-shaped, (40−)45–75(−115) × (3−)5–6(−6.5) μm, with 2 apical hila. *Intercalary conidia* sometimes present, cylindrical to subcylindrical, sometimes curved, occurring in chains of up to 4 conidia, (32−)40−108(−136) × (3−)4−5(−5.5) μm. *Conidia* mostly solitary, sometimes blastocatenate, polymorph [acicular, ovoid to obovoid, cylindrical or obclavate-cylindrical, doliiform, elliptical, Lshaped to sickle-shaped, rarely V-shaped], (14−)25–215(−250) × (2.5−)3–6(−7.5)] µm, obtuse apex, base obconical, truncate at the base, surrounded by circular rim-like structure, 0−12-euseptate, smooth to slightly roughened, light olivaceous brown to brown, hilum thickened and darkened, 1.5−2 μm diam.

Culture characteristics: Colonies slow-growing, reaching a diameter of approximately 6 mm on MEA and 7 mm on PDA after 14 days at 25°C ± 5°C. The colonies were circular in outline with a velvety aerial mycelium. On MEA, the upper surface white and fluffy, while the reverse was black. On PDA, the upper surface ranged from dark gray to black, with a brown to black reverse. On MEA: *Hyphae* (1.5–)2.5–4(–5) μm wide, branched, septate, smooth to slightly roughened and subhyaline to very light olivaceous brown. *Fructification* occurred with the formation of chlamydospores accompanied by seta-like structures. *Setae* branched, septate, smooth to slightly roughened, light brown to dark brown and (2–)2.5–3.5(–4) μm diam. *Chlamydospores* developed in chains, occurring intercalarily and terminally. They were spherical to oval, subhyaline to mid brown, thick-walled, smooth to slightly roughened, (5–)6–17(–23) × (4–)5–7(–8) μm. Germinating chlamydospores were also observed.

Sporulation takes place on agar media supplemented with undefined vegetable peelings. The colonies were whitish gray to smoky black. *Stromata* well developed, hard, irregular, and light olivaceous brown to blackish brown. *Hyphae* branched, septate, smooth-walled, subhyaline to light olivaceous, 2–3 μm wide. *Conidiophores* macronematous, mostly arising in fascicles from the stromata, occasionally solitary, sometimes reduced to a single-celled ampulliform conidiogenous cell, erect to slightly curved, divergent, subcylindrical, basal cell slightly swollen, mostly unbranched, rarely branched, smooth, sometimes slightly roughened, subhyaline to light olivaceous brown, 0−9-septate, thick-walled, (16–)25–70(–90) × (3–)4–5.5(–8.5) μm. *Conidiogenous cells* integrated, terminal as well as intercalary, mono- to polyblastic, cylindrical, conidiogenous loci slightly protuberant, loci thickened and darkened, (1.5−)2–2.5(–3) μm wide. *Conidia* solitary, simple, dry, subhyaline to light olivaceous brown, mostly cylindrical or obclavate-cylindrical, ovoid to obovoid, sometimes curved, smooth-walled, sometimes slightly roughened, thin to thick-walled, tapering toward an obtuse apex, sometimes apical cell swollen, 0−6-septate, constricted at the septa, (13–)18–50(–75) × (3–)4–5.5(–8) μm, base obconically truncated, hilum slightly thickened and darkened, 1.5−2.5 μm diam. *Chlamydospores* developed in chains, occurring intercalarily and terminally, spherical to oval, mostly horizontally but sometimes vertically and obliquely septate, subhyaline to mid brown, thick-walled, smooth to slightly roughened, (6–)8–10(–13) × (7–)9–13(–15) μm. Germinating chlamydospores were also observed.

Specimens examined: INDIA, Uttar Pradesh, Gorakhpur, on *Calotropis procera*, A. S. Moses (Herb. GPU No. KRNC 64, IMI 337033); INDIA, Uttar Pradesh, Gorakhpur, on *Calotropis procera*, Kamal (Herb. GPU No. KK 300, IMI 314694); INDIA, Uttar Pradesh, Gorakhpur, on *Calotropis procera*, C. Gupta (Herb. GPU No. KC-126, IMI 314110); INDIA, Uttar Pradesh, Gorakhpur, *Caltropis procera*, R. K. Verma (Herb. GPU No. KK 213, IMI 300481); INDIA, Uttar Pradesh, Varanasi, 25.2685°N 82.9905°E, on living leaves of *Calotropis gigantea*, 10 September 2024, Sanjay Yadav, MH-BHU 128 (AMH 10781), culture NFCCI 5983, gene sequence GenBank: PV112567 (ITS), PQ816342 (LSU), PV125517 (*Rpb2*); INDIA, Uttar Pradesh, Mirzapur, 25.1337°N 82.5644°E, on living leaves of *Calotropis procera*, 01 December 2024, Soumyadeep Rajwar, MH-BHU 129 (AMH 10782), culture NFCCI 5984, gene sequence GenBank: PV112568 (ITS), PQ816341 (LSU), PV125518 (*Rpb2*).

Notes: The genus *Clypeosphaerella* was established by Guatimosim et al. (2016) with the type species of *Clypeosphaerella sticheri*. This genus is morphologically similar to species of *Mycosphaerella s. lat.* but differs mainly in having a thicker upper wall of the ascomata, which resembles a pseudoclypeus. Moreover, *Clypeosphaerella* is phylogenetically distinct from other mycosphaerella-like fungi (**Figures 1**, **2**) and forms a well-supported clade as determined by Guatimosim et al. (2016).

Three *Clypeosphaerella* species have been reported across the world, namely, *C. calotropidis* (Ellis and Everh.) Videira and Crous (Videira et al., 2017), *C. quasiparkii* (Cheew. et al.) Guatim. et al (Guatimosim et al., 2016), and *C. sticheri* Guatim. et al (Guatimosim et al., 2016). *Clypeosphaerella calotropidis* is the only species in this genus represented by an asexual morph, while the other two species are only known from their sexual morphs.

Basionym of *Clypeosphaerella calotropidis* is *Cercospora calotropidis* Ellis and Everh. Braun (2000a) transferred *Cercospora calotropidis* to the genus *Passalora* based on the morphological observations. He noted that this species was highly variable and exhibited characteristics that were intermediate between several genera, viz., *Passalora* (known for having fasciculate conidiophores and conidia formed singly), *Phaeoramularia* (characterized by conidia formed in chains), and *Mycovellosiella* (identified by secondary superficial hyphae with solitary conidiophores). This intermediate nature justified the transfer to *Passalora*, reflecting its closest alignment with the morphological traits of this genus. Furthermore, Braun (2000a) cited *C. calotropidis* as an example to demonstrate that the genera *Passalora*, *Phaeoramularia*, and *Mycovellosiella* should be merged, a view supported by Crous et al. (2001). A similar diagnostic approach was followed by Wilkinson et al. (2005) for *Passalora calotropidis* (Braun, 2000a) as the phylogenetic analysis based on ITS placed this species in a single-strain lineage closely related to *Pseudocercospora* (Wilkinson et al., 2005).

Based on a multigene analysis (LSU-*Rpb2*-ITS), *Passalora calotropidis* (CBS 129.30) clustered with *Clypeosphaerella quasiparkii* (CBS 123243) with high statistical support, which was found to be closely related to *Pseudocercospora* and separated as a sister lineage of *Distocercospora pachyderma* (CBS 138247) with high statistical support (Videira et al., 2017). Therefore, *Passalora calotropidis* was recombined as *Clypeosphaerella calotropidis* (CBS 129.30). However, this analysis did not incorporate the type species of *Clypeosphaerella*, *C. sticheri*.

Rajeshkumar et al. (2021) introduced the new genus *Pedrocrousiella* based on LSU-*Rpb2* sequence data, which formed a sister lineage to *Distocercospora pachyderma* (CBS 138247) with high statistical support (BI-PP/ML-BS: 1/98). In this analysis, the inclusion of all three species of *Clypeosphaerella*, along with their type species, forming a monophyletic group, suggested a significant finding in their monophyletic evolutionary relationships. However, in the parsimony analysis, the relationship between *Clypeosphaerella sticheri* and other *Clypeosphaerella* species was unresolved. This unresolved relationship might be due to the missing *Rpb2* data for *Clypeosphaerella sticheri* (Rajeshkumar et al., 2021).

*Pteridopassalora* was introduced by C. Nakash. and Crous (Chen et al., 2022). It was established based on LSU-*Rpb2*-ITS sequence data, which clustered closely with the genus *Clypeosphaerella* and formed a sister lineage of *Distocercospora pachyderma* (CBS 138247). The analysis included two species of *Clypeosphaerella*, namely, *C. calotropidis* and *C. quasiparkii*, but not the type species, *C. sticheri*.

Based on both datasets (**Figures 1**, **2**), the type species of *Clypeosphaerella*, *C. sticheri*, is segregated from the other two *Clypeosphaerella* species, *C. calotropidis* and *C. quasiparkii*, which cluster together with the newly generated sequences obtained from the cultures NFCCI 5983 and NFCCI 5984, isolated from *Calotropis* spp., with strong statistical support (BI-PP/ML-BS: 1/96). Consequently, a new genus, *Neoclypeosphaerella*, is introduced to accommodate *C. calotropidis* and *C. quasiparkii*. The significant nucleotide differences between *Clypeosphaerella sticheri* and *Neoclypeosphaerella calotropidis* (ITS: 28 differences with 11 gaps, LSU: 12 differences with 3 gaps) suggest that they do not belong to the same genus and should be maintained as separate, independent genera.

Several cercosporoid fungi have been described from *Calotropis* spp., namely, *Cercospora baroipurensis* Purkay. and Mallik (Purkay. and Mallik, 1978), *Clypeosphaerella calotropidis* (Ellis and Everh.) Videira and Crous (Chupp, 1954; Kamal et al., 1990; U. Braun, 2000a; Wilkinson et al., 2005; Haldar and Ray, 2001; Videira et al., 2017), *Mycosphaerella calotropidis* T.S. Viswan. (Viswan. and Tilak, 1960), *Paracercosporidium microsorum* (Sacc.) U. Braun et al (Videira et al., 2017), and *Pseudocercospora peronosporoidea* (Pat. and Har.) Deighton (Deighton, 1981).

*Cercospora baroipurensis* and *Pseudocercospora peronosporoidea* can be easily distinguished from *N. calotropidis* based on conidial and conidiophore characteristics. In *C. baroipurensis*, the conidia are hyaline, while the conidiophores are colored with thickened and darkened loci and hila. In contrast, *P. peronosporoidea* has both conidia and conidiophores that are colored, without any thickened or darkened loci and hila.

*Clypeosphaerella calotropidis* closely resembles our two collected samples (NFCCI 5983, NFCCI 5984) on *Calotropis* spp., exhibiting several similarities. Both samples exhibit indefinite leaf spots and large circular to irregular blotches merging into black patches. The immersed, subhyaline mycelium produces amphigenous fruiting with stromata filling stomatal openings. Conidiophores, in fascicles, are pigmented, sparingly septate, occasionally branched, and mildly geniculate near the tip, with a blunt or conic apex bearing a conspicuous conidiogenous locus (scar). Conidia are almost straight to slightly curved, cylindrical to obclavate, pigmented, sparingly catenate, septate, with an obconic base and rounded apex. In our collected samples (NFCCI 5983, NFCCI 5984), some additional features were developed only at a very late stage of infection, including the formation of slightly longer mature acicular conidia (up to 250 μm), the occasional development of superficial secondary hyphae, and the catenation of conidia. These features were not observed during the development of the early stages. Our phylogenetic analysis, providing strong statistical support (BI-PP/ML-BS: 1/99), corroborated these morphological findings and confirmed *C. calotropidis* as the type species of a new genus, *Neoclypeosphaerella*.

*Paracercosporidium microsorum* can be clearly distinguished from *N. calotropidis* by its internal hyphae and solitary and cylindrical to obclavate conidia.

The asexual morph of *Mycosphaerella calotropidis* is unknown, making it impossible to compare this name with *N. calotropidis*. Additionally, the absence of molecular sequence data makes it impossible to determine whether it represents the perfect state of *N. calotropidis*. However, this detail is irrelevant in terms of nomenclatural implications for the current case because *Cercospora calotropidis*, the name of the basionym, is much older than *M. calotropidis*. Hence, it only remains open whether the later name is a synonym of *N. Calotropidis* or not.

Based on both datasets, *Marcstadlera* could not be placed within any of the currently described genera of the *Mycosphaerellaceae* (**Figures 1**, **2**) and is positioned as a sister lineage to *Neoclypeosphaerella. Marcstadlera* is represented by its asexual morph and belongs to the cercosporoid group of fungi in *Mycosphaerellaceae* based on both datasets. Many asexual morphs linked to mycosphaerella-like sexual morphs exhibit cercosporoid morphology (Videira et al., 2017). Since sexual morphs are morphologically conserved, genera within *Mycosphaerellaceae* are primarily distinguished based on their asexual morphs (Crous et al., 2009). The type species of *Neoclypeosphaerella*, *N. calotropidis*, is represented by its asexual morph and is morphologically distinct from *Marcstadlera*. *In vivo*, *Neoclypeosphaerella* primarily develops internal mycelium and forms well-developed stromata bearing fascicles of conidiophores that are geniculate-sinuous at the tip, mostly simple, occasionally branched, and septate. The conidiogenous cells are both terminal and intercalary, with slightly protuberant, thickened, and darkened loci. In contrast, *Marcstadlera* exhibits significant morphological differences. *In vivo*, it develops predominantly external mycelium, lacks stromata entirely, and produces conidiophores that are micronematous to semi-macronematous, mononematous, unbranched, and aseptate. These conidiophores arise individually from intercalary or terminal cells of external hyphae and are reduced to conidiogenous cells. The conidiogenous loci (scars), formed on cylindrical or peg-like conidiogenous cells, are unthickened to slightly thickened and darkened. These differences justify the introduction of a new genus, *Marcstadlera*, for this monotypic lineage.

The significant nucleotide differences between *Marcstadlera* and *Neoclypeosphaerella* (ITS: 16, LSU: 14, *Rpb2*: 69) indicate that they cannot belong to the same genus and should be maintained as separate, independent genera.

Although *Marcstadlera* morphologically resembles *Mycovellosiella* species, as both develop secondary superficial hyphae with solitary conidiophores, the two genera are phylogenetically distant (**Figures 1**, **2**). The *Mycovellosiella*-like morphological traits are considered phylogenetically and taxonomically insignificant and appear unreliable (Videira et al., 2017).

In a megablast search of LSU sequences for *Marcstadlera* in NCBI’s GenBank nucleotide database, *Rosenscheldiella brachyglottidis* (PDD 94939) appeared with 96% sequence similarity (508/527) with no gaps. The phylogenetic analysis based on LSU-*Rpb2* (**Figure 1**) placed *R. brachyglottidis* as a sister lineage to *Neoclypeosphaerella*, though with very low statistical support. *Rosenscheldiella brachyglottidis* is represented by its sexual morph, which can be easily differentiated from the closely related sexual morph *N. quasiparkii* (CBS 123243) by forming pseudothecia with fissitunicate asci, which develop externally to the host leaf on small pads of stromatic tissue growing superficially from hyphae that penetrate through the stomata (Sultan et al., 2011). Therefore, the significant morphological differences between *R. brachyglottidis* and *N. quasiparkii* suggest that *R. brachyglottidis* should be tentatively retained in the genus *Rosenscheldiella* rather than being reclassified under *N. quasiparkii*, reflecting uncertainties in the taxonomy of these organisms.

Based on both datasets, it has been confirmed that *Clypeosphaerella*, *Marcstadlera*, *Neoclypeosphaerella*, and *Rosenscheldiella* are distinct genera, forming separate clades (**Figures 1**, **2**).

***Neoclypeosphaerella quasiparkii*** (Cheew. et al.) Raghv. Singh & Sham. Kumar, **comb. nov.**

MycoBank number: MB854799.

≡ *Mycosphaerella quasiparkii* Cheew. et al., *Persoonia* 21, 85 (2008).

= *Clypeosphaerella quasiparkii* (Cheew. et al.) Guatim. et al. *Persoonia* 37, 121 (2016).

Description and illustration: Cheewangkoon et al. (2008).

Notes: Based on dataset 1, *N. calotropidis* and *N. quasiparkii* are clustered together with very low statistical support (BI-PP/ML-BS: 0.90/-) (**Figure 1**). When LSU, ITS, and *Rpb2* are used as barcodes, they provide valuable insights into evolutionary relationships at the species level in *Mycosphaerellaceae* (Chen et al., 2022). In dataset 2, both species are clustered together with high statistical support (BI-PP/ML-BS: 1/96) (**Figure 2**), indicating a close relationship. Therefore, *N. quasiparkii* is accommodated in *Neoclypeosphaerella* along with *N. calotropidis*, despite being represented by different morphs.

## Discussion

Taxonomic challenges within the family *Mycosphaerellaceae* are numerous and arise from both historical classification practices and current methodological limitations. One of the primary issues is the morphological similarity among species—their fruiting bodies and spores are often small, conserved, and difficult to distinguish. This is compounded by morphological convergence across unrelated taxa occupying similar ecological niches, frequently leading to misidentifications. Historically, the genus *Mycosphaerella* served as a “dumping ground” for numerous unrelated species that had minimal or overlapping morphological characteristics. This practice created significant confusion, especially in light of uncertain or incorrect anamorph–teleomorph associations (Crous et al., 2007, Crous et al., 2009; Groenewald et al., 2013). The problem is further compounded by the presence of cryptic species complexes; notably, *Mycosphaerella graminicola* (now *Zymoseptoria tritici*), the causal agent of *Septoria tritici* blotch in wheat, was revealed to consist of several genetically distinct but morphologically similar species (Quaedvlieg et al., 2011).

Modern molecular phylogenetic studies have shown that many traditional genera within *Mycosphaerellaceae* are paraphyletic or polyphyletic, indicating that current classifications often fail to reflect true evolutionary relationships (Videira et al., 2017). However, progress is limited by the lack of molecular data for many species, especially ex-type sequences and material from undersampled regions. Furthermore, different gene regions used in phylogenetic analyses can yield conflicting results (Videira et al., 2017).

Culturing remains a significant challenge, particularly for obligate or poorly sporulating taxa, which restricts the ability to extract DNA, conduct reproductive studies, or perform pathogenicity and fungicide sensitivity tests (Simon et al., 2009; Hunter et al., 2011). In plant disease diagnostics, multiple species can coexist in a single lesion, and high but variable host specificity further complicates accurate identification and understanding of host–pathogen dynamics (Braun et al., 2013, Braun et al., 2015, Braun et al., 2016). Additional complications stem from poorly understood mating systems and the frequent renaming or redefinition of genera, leading to nomenclatural instability (Aylward et al., 2022). This instability hinders effective communication in applied disciplines such as plant pathology and quarantine regulation.

To overcome the challenges posed by the *Mycosphaerellaceae* family, such as taxonomic ambiguity, diagnostic limitations, and fungicide resistance, several modern approaches have been proposed. Molecular phylogenetics and genomics, particularly multilocus sequence typing (MLST) and whole-genome sequencing (WGS), have significantly enhanced the resolution of phylogenetic relationships and revealed cryptic species diversity (Stengel et al., 2022; Menicucci et al., 2025). DNA barcoding using markers like ITS, LSU, *Rpb2*, *EF-1α*, and *β-tubulin* further aids in accurate species identification (Videira et al., 2017; Abdollahzadeh et al., 2020; Bakhshi et al., 2021; Bakhshi and Braun, 2022; Chen et al., 2022). Species boundaries within species complexes were further resolved using sequence data combined with several clades found to be host-specific—supporting the concept of host-driven speciation in the *Mycosphaerellaceae* (Crous et al., 2008; Arzanlou et al., 2009; Li et al., 2020; Ko et al., 2025). These findings highlight the value of a polyphasic approach in fungal taxonomy, integrating molecular, morphological, and ecological data to refine species concepts and improve classification systems (Crous et al., 2013a, Crous et al., 2013b).

Advanced diagnostics like quantitative PCR (qPCR) and loop-mediated isothermal amplification (LAMP) allow rapid pathogen detection, while integrated disease management and genomic-assisted breeding enhance control (Patel et al., 2022). Global collaboration via platforms like MycoBank and GenBank supports data sharing, aiding in managing *Mycosphaerellaceae* challenges (Robert et al., 2013; Hariharan and Prasannath, 2021; Zhang et al., 2023).

This study clarified the phylogenetic placement of the newly introduced genera *Marcstadlera* and *Neoclypeosphaerella* within the *Mycosphaerellaceae* family using an integrative taxonomic approach that combined multigene phylogenetic analysis (based on ITS, LSU, and *Rpb2* barcode genes), morphological characterization, and host/ecological data. The results also confirmed the paraphyly of *Clypeosphaerella* and the polyphyly of *Mycovellosiella*, with isolates clustering into distinct genera consistent with current taxonomic frameworks. These findings support the reliability of DNA-based classification, particularly when morphology alone proves insufficient for distinguishing Mycosphaerellaceaean fungi at the generic level (Videira et al., 2017; Chen et al., 2022).

In the Indian context, fungal diversity is remarkably high, with new species being reported annually. Historically, studies on phytopathogenic fungi related to *Mycosphaerellaceae* in India have primarily relied on morphological data (Singh et al., 2007, Singh et al., 2008, Singh and Kumar, 2011, Singh et al., 2012, Singh et al., 2013, Singh et al., 2014a, Singh et al., 2014b, Singh et al., 2020a, Singh et al., 2020b, Singh et al., 2022; Kamal, 2010; Kumar et al., 2014; Kumar and Singh, 2015, Kumar and Singh, 2016; Singh and Kumar, 2017; Kushwaha et al., 2020; Verma et al., 2023). However, recent investigations (Singh et al., 2020b; Verma et al., 2021a, Verma et al., 2021b; Yadav et al., 2021, Yadav et al., 2022, Yadav et al., 2023) demonstrate a significant shift toward the use of cultures, SEM imaging, and DNA sequencing to support taxonomic conclusions.

Overall, this research contributes to the ongoing revision of *Mycosphaerellaceae* classification using molecular tools, while also uncovering hidden diversity and enhancing our understanding of species and generic boundaries, as well as evolutionary relationships.

## Data Availability

The specimen studied in this work was deposited in the Ajrekar Mycological Herbarium (AMH), Agharkar Research Institute (ARI), Pune and National Fungal Culture Collection of India (NFCCI), Pune, Maharashtra, India. The datasets presented in this study can be found in online repositories. The names of the repository/repositories and accession number(s) can be found below: https://www.ncbi.nlm.nih.gov/genbank/, ITS: PQ012587, PQ013688, PV112567, and PV112568; LSU: PQ012588, PQ013689, PQ816342, and PQ816341; *Rpb2*: PQ034553, PQ034554, PV125517, and PV125518.
